# Epistatic interactions associated with fatty acid concentrations of beef from angus sired beef cattle

**DOI:** 10.1186/s12864-016-3235-8

**Published:** 2016-11-08

**Authors:** L. M. Kramer, M. A. Abdel Ghaffar, J. E. Koltes, E. R. Fritz-Waters, M. S. Mayes, A. D. Sewell, N. T. Weeks, D. J. Garrick, R. L. Fernando, L. Ma, J. M. Reecy

**Affiliations:** 1 Department of Animal Science, Iowa State University, 2255 Kildee Hall, Ames, IA 50011 USA; 2 Department of Animal & Poultry Production/Faculty of Environmental Agricultural Science, Arish University, North Sinai, 45516 Egypt; 3 Department of Animal Science, University of Arkansas, Fayetteville, AR 72701 USA; 4 The Maschhoffs, Carlyle, IL 62231 USA; 5 Department of Mathematics, Iowa State University, Ames, IA 50011 USA; 6 Department of Animal and Avian Sciences, University of Maryland, College Park, USA

**Keywords:** Epistatic interaction, Beef cattle, Fatty acids, Triacylglyceride, Phospholipid

## Abstract

**Background:**

Consumers are becoming increasingly conscientious about the nutritional value of their food. Consumption of some fatty acids has been associated with human health traits such as blood pressure and cardiovascular disease. Therefore, it is important to investigate genetic variation in content of fatty acids present in meat. Previously publications reported regions of the cattle genome that are additively associated with variation in fatty acid content. This study evaluated epistatic interactions, which could account for additional genetic variation in fatty acid content.

**Results:**

Epistatic interactions for 44 fatty acid traits in a population of Angus beef cattle were evaluated with EpiSNPmpi. False discovery rate (FDR) was controlled at 5 % and was limited to well-represented genotypic combinations. Epistatic interactions were detected for 37 triacylglyceride (TAG), 36 phospholipid (PL) fatty acid traits, and three weight traits. A total of 6,181, 7,168, and 0 significant epistatic interactions (FDR < 0.05, 50-animals per genotype combination) were associated with Triacylglyceride fatty acids, Phospholipid fatty acids, and weight traits respectively and most were additive-by-additive interactions. A large number of interactions occurred in potential regions of regulatory control along the chromosomes where genes related to fatty acid metabolism reside.

**Conclusions:**

Many fatty acids were associated with epistatic interactions. Despite a large number of significant interactions, there are a limited number of genomic locations that harbored these interactions. While larger population sizes are needed to accurately validate and quantify these epistatic interactions, the current findings point towards additional genetic variance that can be accounted for within these fatty acid traits.

**Electronic supplementary material:**

The online version of this article (doi:10.1186/s12864-016-3235-8) contains supplementary material, which is available to authorized users.

## Background

Beef cattle producers must contend with the desires of their consumers. Health consciousness amongst consumers has grown as more information and misinformation is publicized about the effects of food components, e.g., fatty acids, on human health. The health risks associated with increased fat consumption are a consideration for many consumers [1, 2] and beef is traditionally thought of as a high fat foodstuff. Producers can select for beef with a healthier fatty acid composition as fatty acid content of beef is highly heritable [3, 4] and genetic markers account for a large amount of this genetic variation [5–9]. However, the impact of non-additive genetic variation on fatty acid variation remains unknown. One of these non-additive genetic factors is the presence of epistatic interactions throughout a genome. While most research focuses on the additive portion of genetic variation, it often cannot account for all the genetic variation predicted. This additional variation may be explained with the identification and characterization of epistatic interactions. Although selection on fatty acid content in cattle with the inclusion of epistatic effects may not be very advantageous due to the breakdown of epistasis during selection [10], the ability to identify the interactions may lead to further understanding of the molecular mechanisms underlying variation in fatty acid content. This study aimed to identify the extent to which epistatic interactions could account for additional genetic variation in fatty acid composition of beef.

## Results and Discussion

Phenotypic summary statistics for TAG and PL fatty acids are reported in Table 1.

**Table 1:** The phenotypic summary statistics for TAG and PL fatty acid traits

Triacylglycerol Fatty Acids				Phospholipid Fatty Acids			
Trait	Mean (*N* = 1721)	SD	CV	Trait	Mean (*N* = 1442)	SD	CV
Saturated Fatty Acids							
SFA	45.8	2.5	0.05	SFA	38.44	5.26	0.14
12:0	0.13	0.19	1.46	12:0	1.4	1.8	1.28
14:0	3.08	0.51	0.16	14:0	3.33	4.55	1.37
16:0	27.35	1.75	0.06	16:0	20.2	3.49	0.17
17:0	1.42	0.39	0.28	17:0	1.75	2.6	1.48
18:0	13.23	1.9	0.14	18:0	9.88	2.51	0.25
22:0	0.02	0.06	2.73	22:0	0.85	1.07	1.26
23:0	0.01	0.06	3.84	23:0	0.17	0.43	2.47
24:0	0.03	0.08	2.83	24:0	0.45	0.66	1.45
Monounsaturated Fatty Acids							
MUFA	51.57	2.56	0.05 MUFA	24.07	7.7	0.32	MUFA
14:1	0.7	0.21	0.3	14:1	0.22	0.54	2.43
16:1	3.82	0.61	0.16	16:1	0.72	0.83	1.15
17:1	1.01	0.33	0.32	17:1	1.21	1.02	0.84
18:1 cis-9	40.26	2.86	0.07	18:1 cis-9	19.31	6.71	0.35
18:1 cis-11	0.11	0.1	0.92	18:1 cis-11	0.08	0.3	3.91
18:1 cis-12	0.3	0.16	0.52	18:1 cis-12	0.04	0.35	9.22
18:1 cis-13	0.11	0.12	1.17	18:1 cis-13	0.09	0.51	5.97
18:1, trans-6/9	0.19	0.45	2.4	18:1, trans-6/9	0.04	0.33	8.29
18:1, trans-10/11	3.69	1.57	0.43	18:1, trans-10/11	0.59	1.18	2.01
18:1, trans-12	0.18	1.15	6.21	18:1, trans-12	0.03	0.19	6.48
18:1, trans-15	1.15	0.36	0.31	18:1, trans-15	1.51	1.68	1.11
Polyunsaturated Fatty Acids							
PUFA	2.63	0.89	0.34	PUFA	37.49	8.72	0.23
18:2	2.01	0.52	0.26	18:2	25.67	6.72	0.26
18:3, n-3	0.16	0.16	0.99	18:3, n-3	0.12	0.71	5.85
20:2	0.08	0.11	1.4	20:2	0.05	0.27	5.32
20:4	0.02	0.08	3.55	20:4	8.4	2.73	0.32
20:5	0.1	0.16	1.68	20:5	0.37	0.56	1.51
22:6	0.06	0.26	4.65	22:6	0.19	0.79	4.14
CLA cis-9 trans-11	0.04	0.09	2.08	CLA cis-9 trans-11	0.06	0.6	10.08
CLA trans-10 cis-12	0.12	0.12	1.03	CLA trans-10 cis-12	0.04	0.29	6.87
n-3	0.33	0.4	1.22	n-3	2.31	2.09	0.91
n-6	2.3	0.63	0.28	n-6	35.18	8.31	0.24
Ratio/Calculation							
n-6:n-3	0.14	0.15	1.05	n-6:n-3	0.07	0.07	1.07
PUFASFA	0.06	0.02	0.36	PUFASFA	1.01	0.31	0.31
MCFA	4.42	0.8	0.18	MCFA	5.21	4.01	0.77
LCFA	95.58	0.8	0.01	LCFA	94.79	4.01	0.04
IA^a^	0.74	0.09	0.12	IA^a^	0.59	0.33	0.56

^a^IA: Index of Atherogenicity

Mean, Standard Deviation (SD), Coefficient of variation (CV) calculated for all 1,721 and 1,442 head of cattle used to identify significant epistatic interactions in triacylglyceride and phospholipids fatty acid traits respectively

Phenotypic summary statistics for Weight Traits are reported in Table 2.

**Table 2:** Summary statistics for weight traits (kilograms)

Trait	Mean (*N* = 2342)	SD	CV × 100
Hot Carcass Weight	332.48	32.66	9.82
Yearling Weight	431.59	82.58	19.13
Weaning Weight	184.86	42.97	23.25

Mean, Standard Deviation (SD), Coefficient of variation (CV) calculated for 2,324 head of cattle used to identify significant epistatic interactions for weight traits

**Fig. 1:**
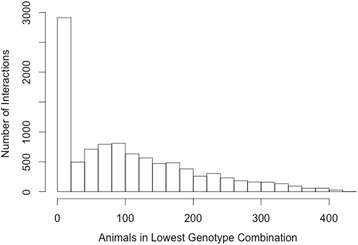
Number of Animals in Lowest Genotype Combination by Interaction Count in TAG Fatty Acids. Histograms of number of animals found in the smallest genotype combination for each TAG interaction against the frequency of their occurrence. All sections left of a given animal cut off represent the number of interactions that would have been detected

**Fig. 2:**
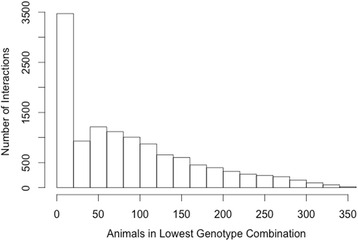
Number of Animals in Lowest Genotype Combination by Interaction Count in PL Fatty Acids. Histograms of number of animals found in the smallest genotype combination for each PL interaction against the frequency of their occurrence. All sections left of a given animal cut off representing the number of interactions that would have been detected

A total of 6,181 and 7,168 SNP by SNP interactions (TAG and PL respectively) had a FDR < 0.05 with data analysis limited to pairs of loci on different chromosomes in which at least 50-animals were represented for every combination of genotypes at the two loci under consideration as in Table 3. A stricter animal number filter was utilized to further restrict the number of identified interactions. The filter were used to determine if increasing genotype combination frequency impacted the number of significant results identified, which may indicate that low frequency genotype combinations were responsible for the generation of spurious significance. The filter was applied by increasing the number of animals present within each genotype combination to 100 animals per genotype combination (data not shown for 5, 10, 20 animal filters). The numbers of significant inter-chromosomal interactions (FDR < 0.05) with either a 50-animal filter or 100-animal filter for both TAG and PL fatty acid fractions are shown in Table 3. The 100-animal filter was the strictest applied and resulted in a steep reduction in the number of significant interactions identified for every fatty acid trait analyzed. This reduction appears to be due to fewer loci being considered as the proportion of significant interactions among all possible interactions were similar at both five and 100 animal filters. As shown in Figs. 1 and 2, the number of interactions identified for a given animal filter continues to decrease as the filter became more stringent, with the total represented by all values to the right of the cutoff. A larger population of animals would be needed to truly represent the real proportion of animals per genotype combination. Once a suitable population is used, a stricter filter (such as the 100-animal filter) could be utilized to further reduce spuriously significant epistatic interactions.

**Table 3:** Number of significant interactions at 50 and 100 animals per genotype combination filters

TAG Fatty acid traits				PL Fatty acid traits			
Trait	FDR	50-Animal Filter	100-Animal Filter	Trait	FDR	50-Animal Filter	100-Animal Filter
Saturated fatty acids							
SFA	3671	249	179	SFA	2595	148	117
12:0	9559	6	5	12:0	1227	39	14
14:0	1017	835	511	14:0	7	7	5
16:0	9559	0	0	16:0	9563	0	0
17:0	164	160	128	17:0	426	406	296
18:0	9449	226	156	18:0	9435	156	94
22:0	9451	1	0	22:0	9458	3	0
23:0	2472	1	0	23:0	9483	1	0
24:0	9569	0	0	24:0	9561	0	0
Monounsaturated Fatty Acids							
MUFA	1	1	1	MUFA	0	0	0
14:1	3	3	0	14:1	9512	0	0
16:1	9440	229	161	16:1	9511	43	27
17:1	265	261	215	17:1	326	315	241
18:1 cis-9	9528	0	0	18:1 cis-9	9567	0	0
18:1 cis-11	922	775	451	18:1 cis-11	3959	3145	1861
18:1 cis-12	1	0	0	18:1 cis-12	0	0	0
18:1 cis-13	9536	7	0	18:1 cis-13	9577	4	0
18:1, trans-6/9	2619	0	0	18:1, trans-6/9	9637	0	0
18:1, trans-10/11	2918	2594	1925	18:1, trans-10/11	1924	1466	933
18:1, trans-12	0	0	0	18:1, trans-12	-	-	-
18:1, trans-15	54	36	26	18:1, trans-15	301	266	180
Polyunsaturated Fatty Acids							
PUFA	9574	0	0	PUFA	9586	0	0
18:2	1	0	0	18:2	1	0	0
18:3, n-3	9723	0	0	18:3, n-3	9622	0	0
20:2	9442	2	2	20:2	9459	0	0
20:4	9455	0	0	20:4	9489	0	0
20:5	9625	0	0	20:5	9634	0	0
22:6	9499	0	0	22:6	9546	1	0
CLA cis-9 trans-11	9559	0	0	CLA cis-9 trans-11	9536	0	0
CLA trans-10 cis-12	53	17	13	CLA trans-10 cis-12	2853	90	52
n-3	9555	0	0	n-3	9588	0	0
n-6	9433	94	58	n-6	9431	155	91
Ratio/Calculation							
n-6:n-3	9556	2	2	n-6:n-3	9601	2	2
PUFASFA	9575	0	0	PUFASFA	9585	0	0
MCFA	8793	337	189	MCFA	8628	461	244
LCFA	8771	341	188	LCFA	8596	460	244
IA^a^	12	4	1	IA^a^	3	0	0
Total	212824	6181	4211	Total	231227	7168	4401

^a^IA: Index of Atherogenicity

Number of significant identified epistatic interactions are displayed from left to right as filtered by FDR < 0.05, 50-animals per genotype combination with FDR < 0.05, and 100-animals per genotype combination with FDR < 0.05 for each fatty acid trait analyzed in both the Triacylclyceride and Phospholipid fatty acid fraction. This sum of the total significant interactions at each of these filters is then provided at the bottom of the table

**Fig. 3:**
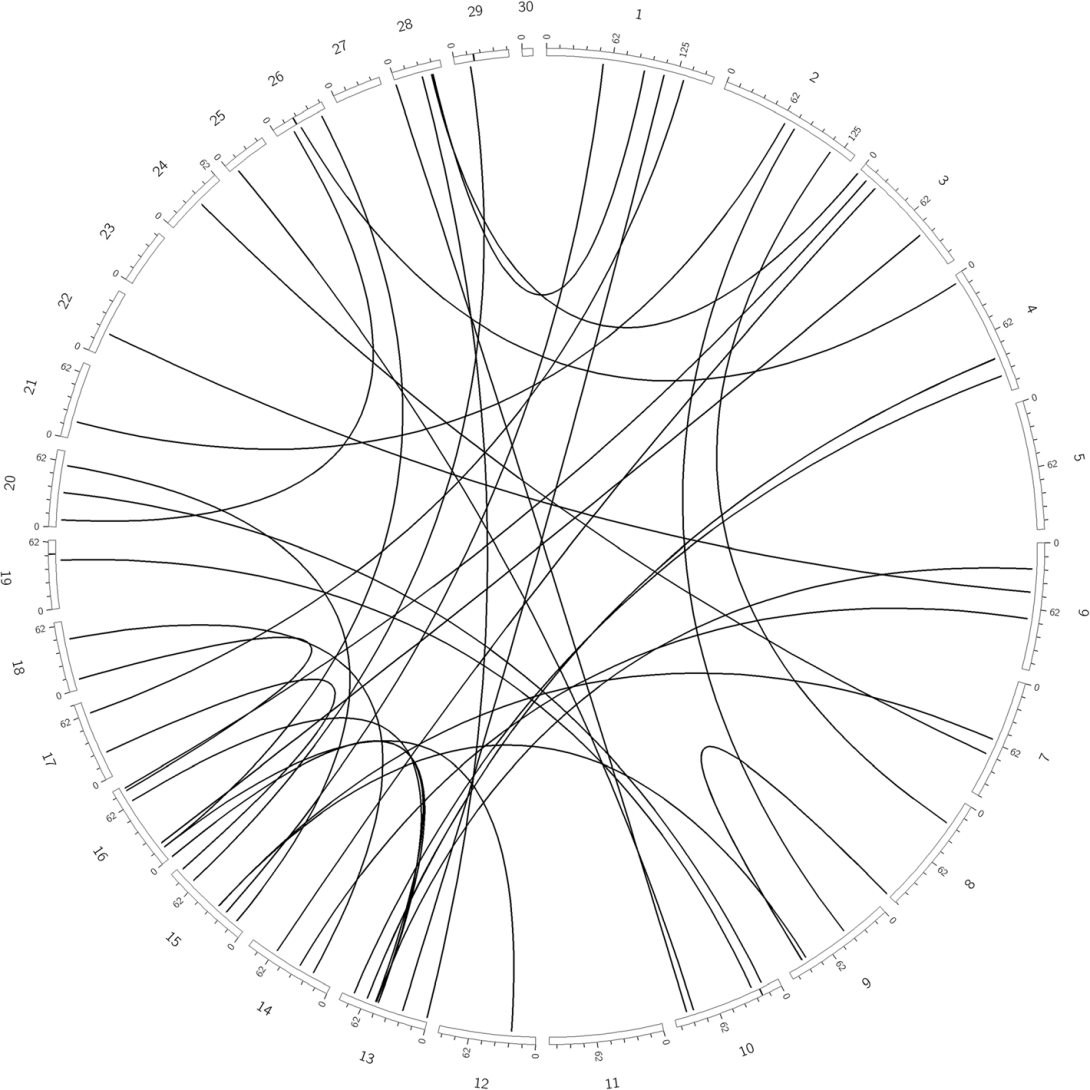
Epistatic interactions plotted for PL fatty acid 16:1 at 50-animals per genotype. 265 total interactions plotted. *Black lines*: Additive x Additive interactions. *Red lines*: Additive x Dominance interactions. *Blue lines*: Dominance x Dominance interactions. Chromosomes ordered around outside with distance ticks every 12.5 MB. *Black marks* represent previously discovered 1 MB regions accounting for large amounts of additive variance

The observed modes of inheritance of the identified epistatic interactions included all the possible combinations, namely additive-by-additive (AA), additive-by-dominance or dominance-by-additive (AD), and dominance-by-dominance (DD). The fatty acids in Table 4 are sorted by fatty acid fraction (TAG and PL) as well as the four fatty acid categories (SFA, MUFA, PUFA, and Ratio/Calculation) at a filter of FDR < 0.05 and 50-animals per genotype combination. TAG and PL fatty acid fractions had similar proportions of epistatic modes of inheritance (AA, AD, DD) between the four fatty acid categories. Dominance-by-dominance was the least commonly observed (summed across all fatty acid traits) mode of inheritance within the TAG fatty acids (0.06 % of total significant interactions) as well as within PL fatty acids (0.01 % of total significant interactions. For both fatty acid fractions (TAG and PL) additive-by-additive interactions made up the largest proportion of significant interactions, with 98.53 and 98.45 % respectively (e.g., for PL 16:1 represented in Fig. 3). Polyunsaturated fatty acids made up the smallest proportion of significant interactions for both the TAG (1.83 %) and PL (3.43 %) fraction. Interactions by type (AA, AD, or DD) were similar in levels and proportions between TAG and PL fractions, although a few traits exhibited different compositions of interactions (e.g., 14:0 – predominantly AA in TAG fraction while nearly non-existent in PL fraction). Visualization of additional fatty acids in both fractions can be found in the Additional file 1, all charted at 50-animals per genotype combination.

**Table 4:** Significant interactions by epistatic type at 50-animals per genotype combination

Triacylglycerol Fatty Acids					Phospholipid Fatty Acids				
Trait	AA	AD	DD	Total Interactions	Trait	AA	AD	DD	Total Interactions
Saturated Fatty Acids									
SFA	226	23	0	249	SFA	131	16	1	148
12:0	6	0	0	6	12:0	34	5	0	39
14:0	817	16	2	835	14:0	7	0	0	7
16:0	0	0	0	0	16:0	0	0	0	0
17:0	158	2	0	160	17:0	399	7	0	406
18:0	223	3	0	226	18:0	155	1	0	156
22:0	0	1	0	1	22:0	2	1	0	3
23:0	0	1	0	1	23:0	0	1	0	1
24:0	0	0	0	0	24:0	0	0	0	0
Monounsaturated Fatty Acids									
MUFA	1	0	0	1	MUFA	0	0	0	0
14:1	3	0	0	3	14:1	0	0	0	0
16:1	224	5	0	229	16:1	43	0	0	43
17:1	260	1	0	261	17:1	313	2	0	315
18:1 cis-9	0	0	0	0	18:1 cis-9	0	0	0	0
18:1 cis-11	769	6	0	775	18:1 cis-11	3110	35	0	3145
18:1 cis-12	0	0	0	0	18:1 cis-12	0	0	0	0
18:1 cis-13	7	0	0	7	18:1 cis-13	4	0	0	4
18:1, trans-6/9	0	0	0	0	18:1, trans-6/9	0	0	0	0
18:1, trans-10/11	2569	23	2	2594	18:1, trans-10/11	1449	17	0	1466
18:1, trans-12	0	0	0	0	18:1, trans-12	-	-	-	-
18:1, trans-15	36	0	0	36	18:1, trans-15	265	1	0	266
Polyunsaturated Fatty Acids									
PUFA	0	0	0	0	PUFA	0	0	0	0
18:2	0	0	0	0	18:2	0	0	0	0
18:3, n-3	0	0	0	0	18:3, n-3	0	0	0	0
20:2	2	0	0	2	20:2	0	0	0	0
20:4	0	0	0	0	20:4	0	0	0	0
20:5	0	0	0	0	20:5	0	0	0	0
22:6	0	0	0	0	22:6	1	0	0	1
CLA cis-9 trans-11	0	0	0	0	CLA cis-9 trans-11	0	0	0	0
CLA trans-10 cis-12	15	2	0	17	CLA trans-10 cis-12	73	17	0	90
n-3	0	0	0	0	n-3	0	0	0	0
n-6	92	2	0	94	n-6	150	5	0	155
Ratio/Calculation									
n-6:n-3	2	0	0	2	n-6:n-3	2	0	0	2
PUFASFA	0	0	0	0	PUFASFA	0	0	0	0
MCFA	336	1	0	337	MCFA	460	1	0	461
LCFA	340	1	0	341	LCFA	459	1	0	460
IA^a^	4	0	0	4	IA^a^	0	0	0	0
Total	6090	87	4	6181	Total	7057	110	1	7168

^a^IA: Index of Atherogenicity

Number of Additive-by-Additive (AA), Additive-by-Dominance and Dominance-by-Additive (AD), Dominance-by-Dominance (DD) significant epistatic interactions by type for Triacylclyceride and Phospholipid fatty acid traits by trait category (Saturated Fatty Acid [SFA], Monounsaturated Fatty Acid [MUFA], Polyunsaturated Fatty Acid [PUFA], Ratio/Calculation). All interactions are post FDR <5 % correction

The distribution of identified epistatic interactions across fatty acid traits is of more interest. The number of interactions that met our minimum filter criteria (inter-chromosomal, 50-animals per genotype combination, FDR < 0.05) varied greatly between traits within each fatty acid fraction, and even between fractions for the same trait. For example, there were no significant (FDR < 0.05) interactions detected for 17 TAG fatty acids at 50-animals per genotype combination, while 2,594 significant (FDR < 0.05) interactions were associated with fatty acid 18:1t10pt11. No significant (FDR < 0.05) interactions were detected for 17 PL fatty acids at 50-animals per genotype combination, but 3,145 significant (FDR < 0.05) interactions were associated with PL fatty acid 18:1c11. This range in number of significant interactions identified for each trait was not confined to any specific fatty acid isoform or category (SFA, MUFA, PUFA, Ratio/Calculation) for either of the two fractions of fatty acids. Many exhibited a similar number of significant interactions in both TAG and PL fatty acid fractions, although there are exceptions such as fatty acids 14:0 which had a greater than 10-fold difference in number of interactions between TAG and PL fatty acids.

Polyunsaturated fatty acids had the lowest number of significant (FDR < 0.05) interactions of all fatty acid types, in that all 11 fatty acids had less than 100 significant (FDR < 0.05) epistatic interactions for the TAG fraction, and 10 out of 11 fatty acids for the PL fraction. Only n6 fatty acids (the sum of all omega-6 fatty acids) had more than 100 significant (FDR < 0.05) interactions, and this was only in the PL fraction. Over half of the significant (FDR < 0.05) interactions associated with polyunsaturated fatty acids were with n6 fatty acids for both TAG and PL fractions.

Among 18:1-cis fatty acids, there was a large range in the number of observed significant interactions. Triacylglyceride fatty acids 18:1c9 and 18:1c12 were not significantly (FDR < 0.05) associated with any epistatic interactions while fatty acids 18:1c11 and 18:1c13 were associated with 775 and 7 significant (FDR < 0.05) epistatic interactions, respectively. The same trend was observed in the PL fatty acid fraction, fatty acids 18:1c9 and 18:1c12 having no significant epistatic interactions, while 18:1c11 and 18:1c13 had 3,145 and 4 significant (FDR < 0.05) epistatic interactions respectively. This was also seen within the 18:1-trans fatty acids, which had numbers of interactions ranging from zero for 18:1t6t9 and 18:1 t12, up to 2,594 interactions for 18:1t10t11 (FDR < 0.05) for the TAG fatty acid fraction. Within the 18:1-trans fatty acid isoforms of PL fatty acid fraction, 18:1t6t9 was association with zero epistatic interactions, 18:1 t15 was significantly associated with 266 epistatic interactions (FDR <0.05), while 18:1t10p11 was significantly associated (FDR < 0.05) with 1,466 epistatic interactions.

Variation in the number of associated significant epistatic interactions within a single isoform suggests that a multitude of genomic regions control some fatty acid isoforms. Understanding exactly what controls exist within these genomic regions, however, would require a larger population of animals and is therefore not within the bounds of this study. This same range of significant interaction numbers can be seen in the saturated fatty acids. In the TAG fatty acid fraction, the number of significant interactions (FDR < 0.05) ranged from zero (eg. 16:0) up to 835 (14:0), with a range of interactions in-between for the other fatty acid traits. In the PL fatty acid fraction, the number of significant interactions (FDR < 0.05) ranged from zero (eg. 16:0) to 406 (18:1c11).

In the TAG fatty acid fraction, 19 of the 37 traits examined had no significant interactions detected (FDR < 0.05, 100-animals per genotype). Polyunsaturated fatty acid (PUFA) was the only fatty acid category (Saturated: SFA; Monounsaturated: MUFA; Polyunsaturated: PUFA; Ratio/calculation) which did not contain any fatty acid traits with greater than 100 significant interactions after application of the FDR (<0.05) and the 100-animal filter, while the other three categories (SFA, MUFA, Ratio/Calculation) all contained multiple traits with over 100 significant interactions after application of the 100 animal filter. Fatty acid 18:1t10t11 was the sole fatty acid which still had over 1,000 significant interactions after the most conservative filter was applied, with a total of 1,925 significant interactions (FDR < 0.05). For the PL fatty acid fraction, 21 of the 36 fatty acid traits had zero significant interactions (FDR < 0.05, 100-animals per genotype), and just like the TAG fatty acid fraction, PUFA was the only category that did not have any traits with over 100 significant interactions. The fatty acid trait 18:1c11 had the largest number of significant epistatic interactions at 1,861 after both FDR < 0.05 and the 100-animals per genotype filter. Differences in the number of identified significant interactions for each fatty acid trait may be related to the biological role of the fatty acid. Those fatty acids essential to tissues or biological processes may have more epistatic interactions (similar to the concept of genetic redundancy) to ensure their functionality. Fatty acids with less important biological roles or more commonly available through the environment may be under less or no genetic control under normal conditions. The presence of few significant PUFA interactions compared to MUFA interactions is an example of the importance of specific fatty acids. Oleic acid (18:1) is one of the most common MUFA found in animals, and is a building block for the production of linoleic acid (18:2). For this reason the production of oleic acid may be more important to maintain due to its use in the production of more complex fatty acids.

Due to the detection of many significant epistatic interactions, other phenotypic weight traits also collected on these cattle were analyzed utilizing the same methodology to determine if the observed modes of inheritance were unique to fatty acid traits. The body traits used were hot carcass weight, yearling weight, and weaning weight. An FDR filter of < 0.05 and a minimum of 50-animals per genotype combination was used to determine and identify significant SNP interactions. For each of the weight traits, fewer than five significant interactions were observed (WW – 1, YW – 3, HCW - 0) [Table 5]. The small number of interactions identified may be due to selection on these traits, as this would work on additive genetic variance and break down epistatic genetic variance [10]. Strong correlations between fatty acid traits and carcass traits under selection may be one explanation for the variability in number of identified interactions, but this is beyond the scope of the current research.

**Table 5:** Significant interactions by epistatic type at 50-animals per genotype combination

Trait	AA	AD	DD	Total Interactions
Weight Traits				
WW	1	0	0	1
YW	2	1	0	3
HCW	0	0	0	0
Total	3	1	0	4

Number of Additive-by-Additive (AA), Additive-by-Dominance and Dominance-by-Additive (AD), Dominance-by-Dominance (DD) significant epistatic interactions by type for Weight traits Weaning Weight (WW), Yearling Weight (YW), and Hot Carcass Weight (HCW). All interactions are post FDR <5 % correction

**Fig. 4:**
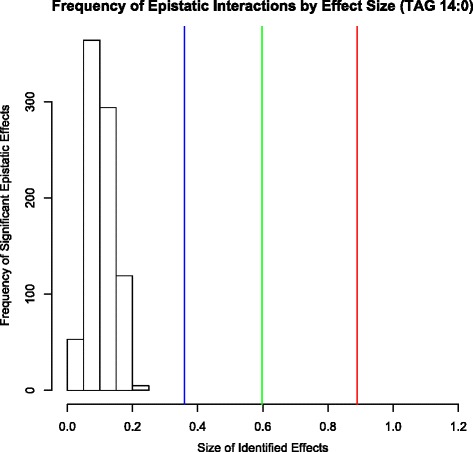
Epistatic effect size frequency compared to individual SNP window effects. The frequency of epistatic genotype combination substitution effects by effect size is plotted for Epistatic interactions in TAG Fatty Acid 14:0. They are compared to the three largest individual SNP window effects for Fatty Acid 14:0 with a minor/major allele frequency 0.4/0.6 represented with vertical lines *red* (Chr:Mb start – 19:51), *green* (Chr:Mb start – 29:18), and *blue* (Chr:Mb – 10:19) identified in Saatchi et al. [6]

It became apparent a few regions of the genome were potential regions of regulatory control while visualizing the genomic regions of significant inter-chromosomal interactions. These include regions like the beginning of chromosomes 1, 18, 20, 21, 24, 28, the middle of chromosomes 3, 8, 10, and the end of chromosomes 2, 4, 14, 17, 21, 28, 29. These locations tended to contain a large number of the identified epistatic interactions within relatively small regions on their respective chromosome. These regions tend to be maintained across TAG and PL fractions of fatty acids, although there are some inconsistencies (fatty acid 12:0 regions in TAG fraction versus relatively low activity in the PL fraction). A total of 206 regions were evaluated with Generic Gene Ontology Term Finder, and terms with an FDR < 0.05 were investigated for relevance to fatty acids. A total of 118 Genes were found associated with the 206 regions. Eleven genes were found associated with GO terms related to lipid metabolism. GO terms found included: Lipid metabolic process, very long-chain fatty acid metabolic process, unsaturated fatty acid metabolic process, and membrane lipid biosynthetic process. Of the 11 genes associated with the GO terms, three (ALOX5, PIK3IP1, and PLA2G2F) were found within the regions analyzed, and also associated with regulatory processes. The relatively small number of genes identified may be due to the presence of LD in the regions,

The effect size of detected significant epistatic interactions were compared to the effect size of the three largest windows identified in Saatchi et al.[6]. Three of the largest 1 mb windows had effects reported by Saatchi et al. for fatty acid trait 14:0 which were compared to the effect sizes of significant TAG epistatic results (Fig. 4) for the same trait. Absolute effect values for epistatic interactions were plotted against the three largest windows by effect size identified in the aforementioned publication given a minor/major allele frequency of 0.4/0.6 (to allow for conservative estimations of effect size). The location of these single window markers relative to SNP interaction effect sizes in Fig. 4 indicated that few if any of the epistatic interactions were of comparable effect size to those explained by the additive effects of SNP windows. When calculating the amount of genetic variance accounted for by the epistatic interactions with GenSel [11], it was determined that the total variance accounted for was already accounted for by single SNP variance. Each epistatic window included in the BayesC analysis resulted in 0.00 % additional genetic variation accounted for with low posterior probabilities of inclusion. The lack of any epistatic interactions being included in the model is likely due to variation from epistasis being accounted for in the single SNP models. Based on this result it would appear that epistatic interactions do not contribute much to additional genomic variation for fatty acid traits. A potential reason for this could be the fact that most of the epistatic interactions can be explained as linear combinations of SNPs, and so are already taken into account in methods such as used by Saatchi et al. [6] that simultaneously fit the effects of many loci. Research has found that although epistatic interactions can be identified for various traits in beef cattle, they are usually non-informative due to difficulty in accurately estimating them, and that their effects do not improve the accuracy of prediction [12].

**Fig. 5:**
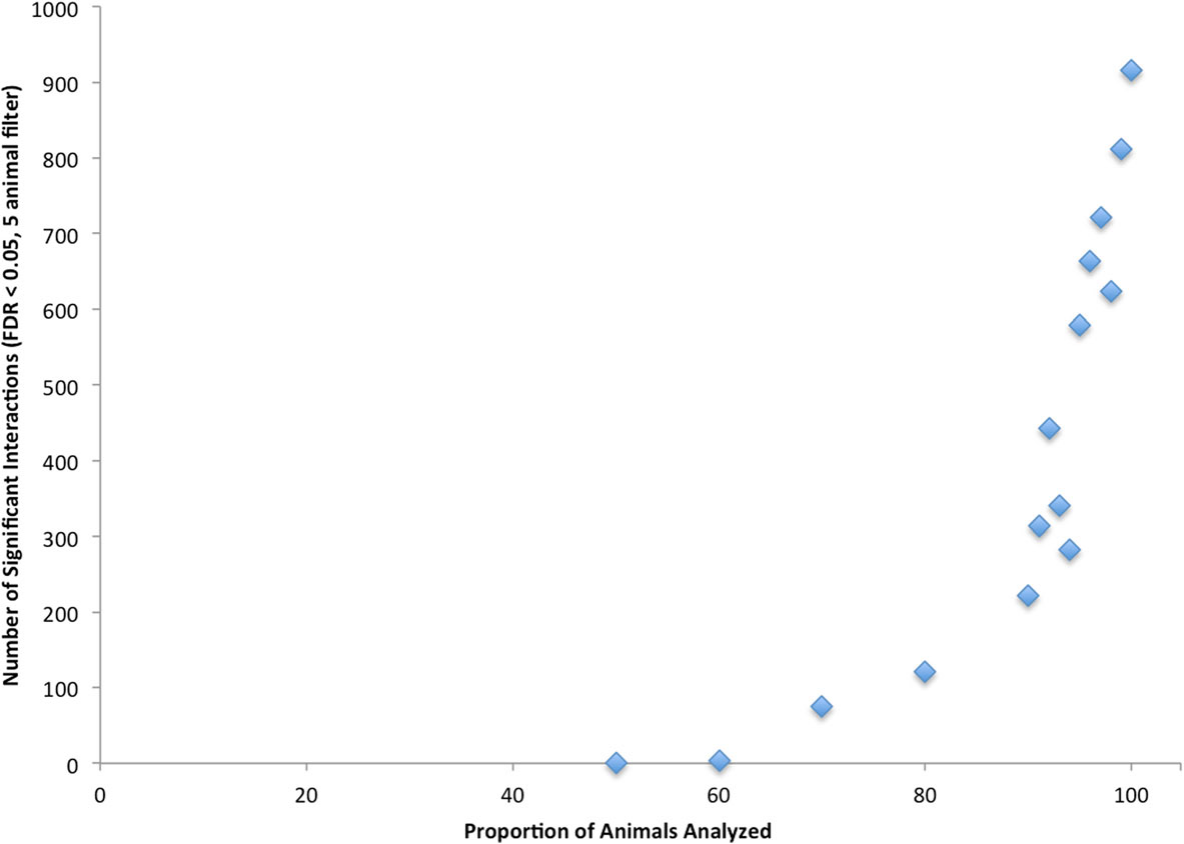
Number of Significant Epistatic Interactions identified with a random proportion of total animals. The proportion of animals out of the total number of animals was randomized each time, and was done at 50, 60, 70, 80, 90, 91–100 %. This analysis was performed using the TAG 18:1c11 fatty acid trait. Filters used: FDR (<0.05), 5 animals per genotype combination; points represent number of interactions detected at a given proportion of animals used

Evaluation by sampling a random proportion of the total population studied and retesting for significant interactions yielded results similar to what was expected based on other studies. As illustrated in Fig. 5, there was a rapid decrease in total number of significant interactions identified as the population size decreases. The number of significantly identifiable interactions had dropped nearly five-fold when 90 % of the original population was used. Unique significant epistatic interactions were not conserved as the proportion of individuals changed, indicating a lack of power to identify true significant interactions. This remained true even after repeating the 50 % proportion 5 times in a row. The significant interactions at the 100-animal filter did not reappear when the subset of individuals was re-randomized. The sharp increase in number of interactions as we approached the total number of animals used in this study indicated that the population size must be greater than that currently available in order to effectively validate any results through cross validation approaches. There is no way to know the inflection point at which additional animals yield less new information based solely on the current results [12, 13]. An increase in the number of animals would be required to appropriately carry out a training-validation study, due to the number of animals needed to have each epistatic interaction genotype combination at a higher frequency.

## Conclusions

Many epistatic interactions were identified in two fractions of fatty acid content, triacylglyceride and phospholipid, for more than 35 fatty acid traits within each of these fractions. Interactions were significant if they met two filter requirements: a false discovery rate of <0.05, and an animal filter of at least 50-animals per genotype combination. An additional filter was applied at 100 animals per genotype combination. A total of 6,181 interactions were identified as significant for the TAG fraction, and 7,168 interactions significant for the PL fraction at a 50-animal per genotypic combination filter. At the 100-animal filter there were still 4,211 and 4,401 significant interactions for the TAG and PL fractions respectively. The number of significant interactions per fatty acid trait varied greatly. This may indicate that some fatty acid traits may need varying amounts of genetic redundancy or control for the creation of fatty acids critical to fitness. Visualization of significant epistatic interactions revealed many regions of potential regulatory control across multiple chromosomes within both the TAG and PL fractions. These regions did not match previously identified windows that accounted for high-genomic variation [6], but they did map to regions that contained relevant genes for fatty acid metabolism. In particular, three genes associated with lipid metabolism GO terms (ALOX5, PLA2G2F and PIK3IP1) were found to exist in regions with large numbers of epistatic interactions. This likely indicates that many interactions involved in a single region may be picking up a single effect, with the SNPs of the region being in LD with the causal mutation(s). Other regions may be mapping to QTL in LD with lipid metabolic controlling genes throughout the genome.

Although large numbers of interactions were identified, validation of these interactions proved to be a difficult task. Reducing the number of animals in order to perform a training-validation test proved futile as even when dropping the number of animals by 10 %, there appeared a steep decline in the number of identifiable interactions. Accordingly, unique epistatic interactions were not conserved during validation between proportions of the population. Despite this, the presence of so many interactions at even a filter of 100-animals per genotype combination indicates that some of the identified interactions may be real. Nevertheless, our access to this large population of animals provided the unique opportunity to set a higher bar for requirements when analyzing epistatic interactions than have been used in the past. Comparison of epistatic interaction effect levels compared to SNP windows with previously identified QTL effects helps validate the possibility that the epistatic effects identified in this study may be real, indicating that some have nearly as large an effect as the additive QTL effects of the SNP windows themselves. This research has shown that while limitations still exist with identifying epistatic interactions, there are ways to help minimize these shortcomings as well as further identifying what needs to be accomplished to overcome the deficiencies.

## Methods

### Population

A subset (1,721 head for TAG fatty acid, 1,442 for PL fatty acid, 2,342 for body traits) of the population described in Garmyn et al. [14] was used in this study.

### Phenotypic data

The triacylglyceride (TAG) and phospholipid (PL) fraction of the longissimus dorsi was analyzed for fatty acid content. Fatty acid data was collected and characterized as described in Saatchi et al. [2]. Briefly, total lipid quantities were extracted from 1.27-cm steaks using a chloroform and methanol mixture. The TAG and PL fractions were separated via thin-layer chromatography, and individual lipid spots were characterized via gas chromatography. The PL portion was calculated by measuring total phosphorus amount from the total lipid portion [15]. The index of atherogenicity was calculated as a ratio of C14:0 and C16:0 fatty acids over the sum of both MUFA and PUFA [16].

$$ AI = \frac{4*C14:0+C16:0}{\Sigma MUFA + \Sigma PUFA} $$

### Genotype data

The BovineSNP50 BeadChip (Illumina, San Diego, CA) was used to perform SNP genotyping at GeneSeek (Lincoln, NE). SNPs were curated at a call rate of 0.95. SNP genotypes were split into individual chromosome files, which were reformatted with in-house R and shell scripts to calculate minor allele frequencies (MAF) for each SNP in order to allow for proper usage with EpiSNPmpi. All SNPs with MAF less than or equal to 0.1 were removed from the study due to lack of power to detect plausible epistatic interactions, resulting in a total of 45,219 SNPs. All SNP markers were assigned a UMD3.1 bovine genome build position.

### Genotype DataAnalysis of pairwise SNP epistatic interactions

EpiSNPmpi was used to statistically analyze the fatty acid traits and identify epistatic interactions [17, 18]. Covariate of carcass contemporary group and lab sampling contemporary group, as well as pedigree and sex data were used in the model. Individuals with unknown sires were assigned a dummy Sire ID unique to them. EpiSNPmpi took each SNP locus in the genotype file, and compared it to every other SNP with a statistical test for four pairwise epistatic effects: additive-by-additive (AA), additive-by-dominance or dominance-by-additive (AD), and dominance-by-dominance (DD). For each fatty acid trait, the top 10,000 pairwise SNP interactions based on significance were obtained. Interactions (AA, AD, and DD) between two SNP on the same chromosome were not considered to limit the chance that detected interactions were markers for a haplotype effect containing a single QTL. The remaining SNP interactions were further filtered such that only those with at least 5 animals in every genotype combination were considered. Thus, any interaction that consisted of even a single genotype combination containing less than 50 observations in 1,721 total (2.9E-02) for TAG and 50 in 1,442 total (3.46E-02) for PL was removed from consideration. An even more stringent 100 animal per genotype filter was also applied to attempt to remove spurious epistatic associations (Table 3). Filters based on 5, 10, and 20 animals per genotype combination are included in Additional file 1.

### Genotype DataMultiple testing correction

A false discovery rate (FDR) correction (<0.05) was applied to each trait to correct for multiple testing. Briefly, interactions were sorted on their nominal p-values, and then FDR values were calculated by the formula.

$$ \frac{\left(P- value\right)\left(\#\  of\  total\  calculations\right)}{Rank\  of\  the\  interaction} $$

Only interactions that fell within FDR <0.05 were reported.

### Window analysis

A BayesC model was used in GenSel to fit SNP and epistatic effects [19]. To calculate the amount of genetic variance that could be accounted for by epistatic interactions, markers were created to represent the possible genotype combinations for a given interaction. Gensel [11] was run with each window representing an entire epistatic interaction (1 window = total effect of a single epistatic interaction). Each window was repurposed as a single marker and added to the map file with a dummy megabase region. GenSel was then run to compare single SNP effects to the addition of interaction SNP effects. Total genetic variation accounted for was compared between two models (SNPs + window markers vs single SNP model [6]) and used to determine how much additional variation could be accounted for through the addition of epistatic interactions.

### Validation

Animals were randomly sampled to obtain different proportions of the total animal population, and then all SNPs were retested with EpiSNPmpi with an FDR (<0.05) and 50 animals per genotype combination filter (Fig. 5). The number of identified interactions was plotted against proportion of animals used to identify the number of significantly identified epistatic interactions within each given proportion. Identified epistatic interactions were compared between each proportion to determine conserved interactions. Individuals used in the 50 % proportion were randomly divided into two groups, with half of the remaining individuals added to each of the two groups as well. This resulted in two groups of 50 % the total animals used. This process was repeated five times to see if the same interactions at a 100 animal filter would reappear in the repeated process.

### Gene enrichment analysis and GO term identification

Regions of potential epistatic regulation were determined by requiring 10 or more interactions to lie within a single megabase region. Locations and windows were determined from the UMD3.1 *Bos taurus* genome build to obtain the chromosome positions [20]. The resulting chromosome positions were combined and a chromosomal window was examined to either side of these SNP locations. Regions were defined as half a megabase upstream and downstream from the central location of the SNPs identified. Regions were put in ENSEMBL Biomart, and the associated Gene Name was obtained for all genes present in the regions. These genes were then run through the Generic Gene Ontology Term Finder [21], and GO terms were obtained by requiring an FDR < 0.05. Terms associated with fatty acid metabolic processes were used to identify genes associated with fatty acid epistasis.

## Additional file

Additional file 1: Supplemental Data (TAGFAinteractions.xlsx, PLFAinteractions.xlsx, and CarcassInteractions.xlsx) and Figures (Circos Plots). (ZIP 22719 kb)

## Abbreviations

TAG: Triacylglyceride
PL: Phospholipid
SFA: Saturated Fatty Acid
MUFA: Monounsaturated Fatty Acid
PUFA: Polyunsaturated Fatty Acid
GO: Gene Ontology
SNP: Single Nucleotide Polymorphism
MAF: Minor Allele Frequency
