# Supplementary figures and images for: Epistatic interactions associated with fatty acid concentrations of beef from angus sired beef cattle
Source: BMC Genomics. 2016 Nov 8;17:891. doi: 10.1186/s12864-016-3235-8 (PMC5100273; doi:10.1186/s12864-016-3235-8)

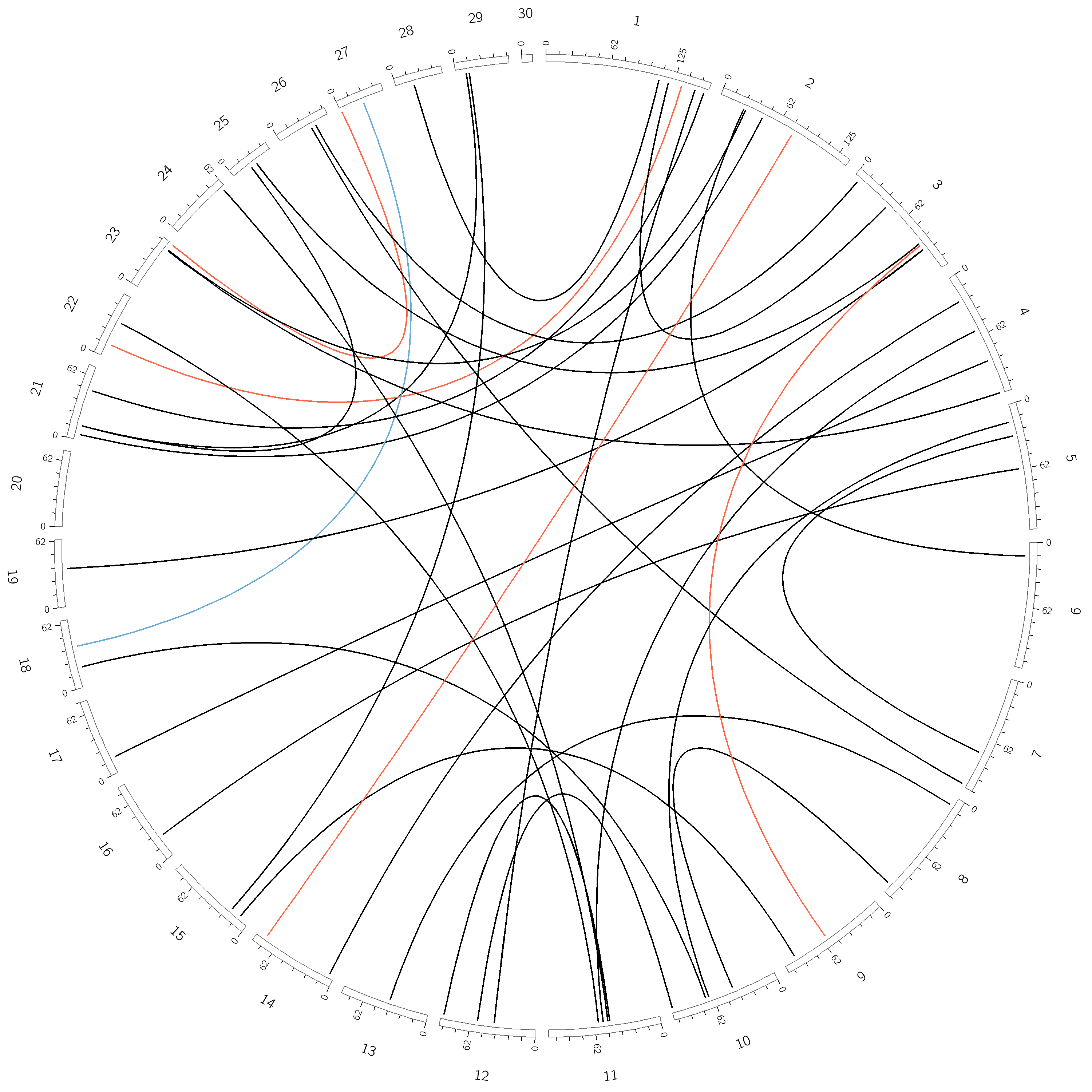

Supplement: Additional file 1: — Supplemental Data (TAGFAinteractions.xlsx, PLFAinteractions.xlsx, and CarcassInteractions.xlsx) and Figures (Circos Plots). (ZIP 22719 kb) [file 12864_2016_3235_MOESM1_ESM.zip › PL12.png]

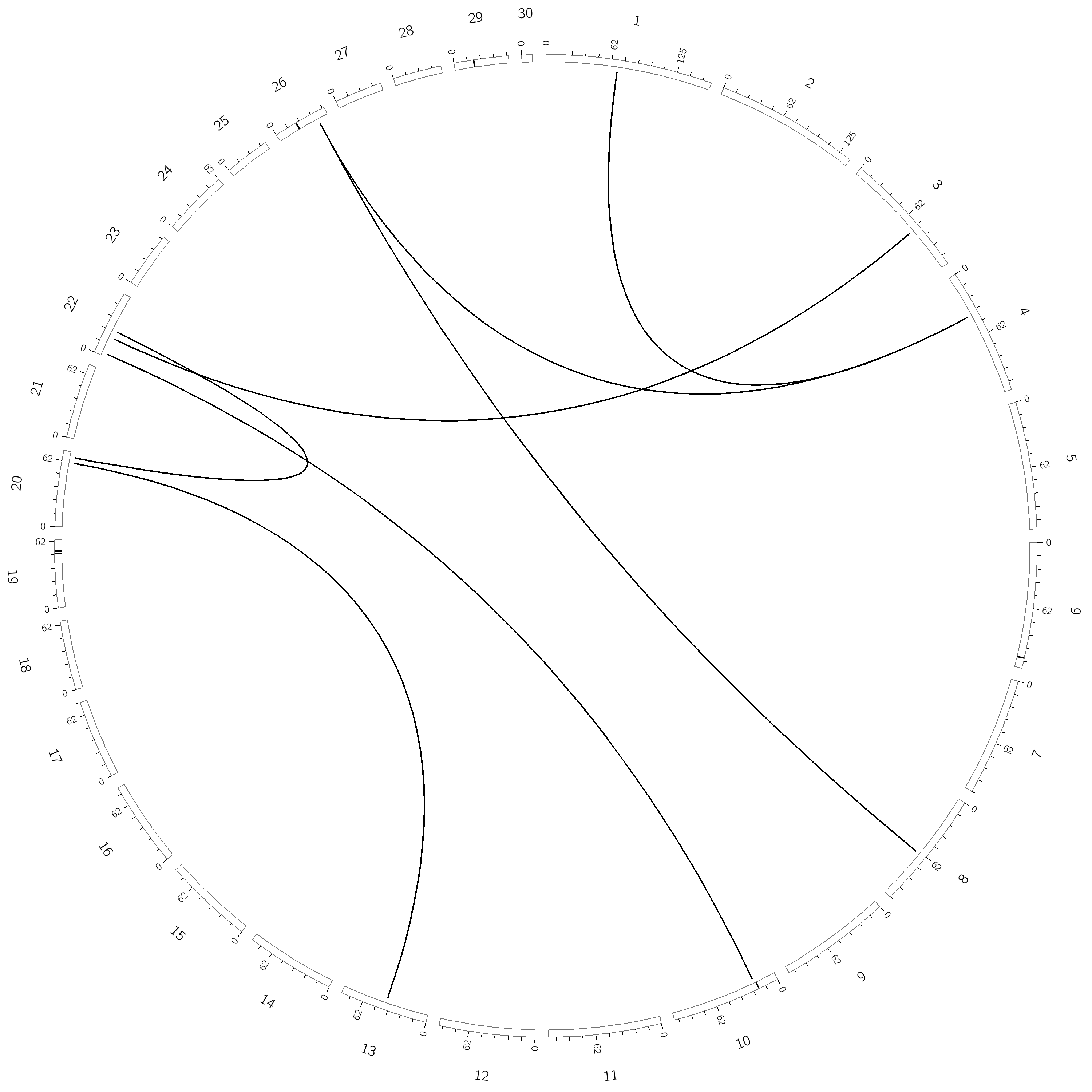

Supplement: Additional file 1: — Supplemental Data (TAGFAinteractions.xlsx, PLFAinteractions.xlsx, and CarcassInteractions.xlsx) and Figures (Circos Plots). (ZIP 22719 kb) [file 12864_2016_3235_MOESM1_ESM.zip › PL14.png]

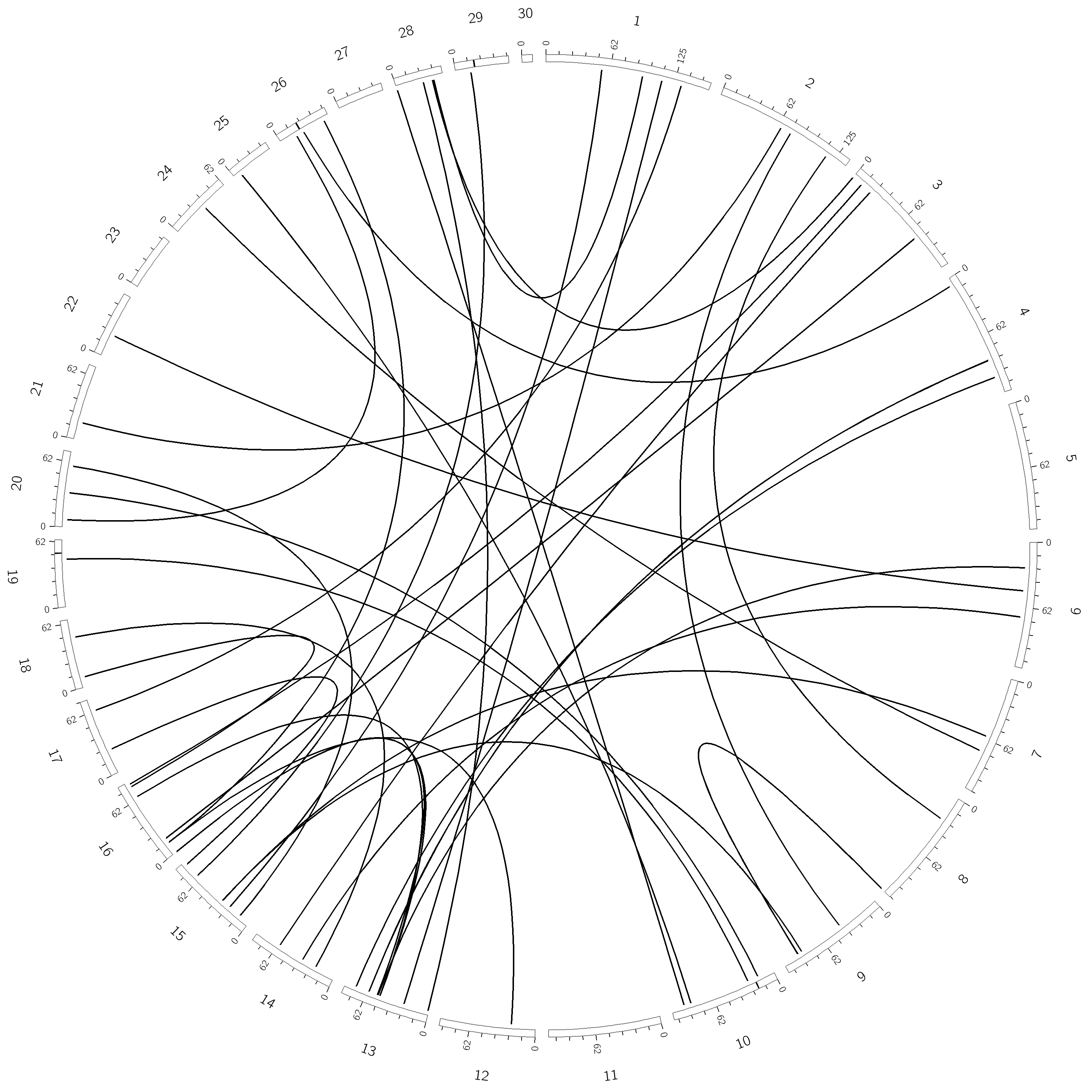

Supplement: Additional file 1: — Supplemental Data (TAGFAinteractions.xlsx, PLFAinteractions.xlsx, and CarcassInteractions.xlsx) and Figures (Circos Plots). (ZIP 22719 kb) [file 12864_2016_3235_MOESM1_ESM.zip › PL161.png]

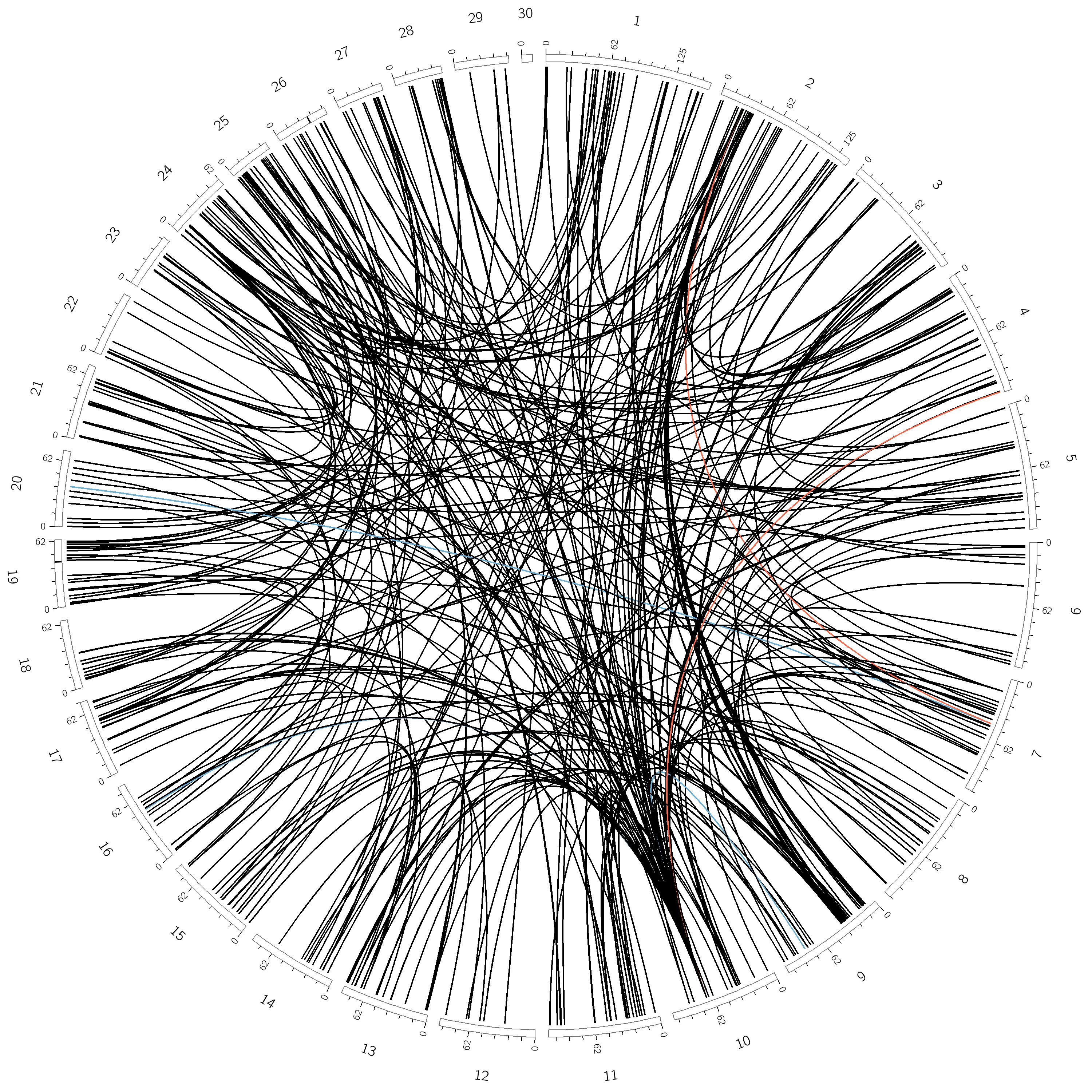

Supplement: Additional file 1: — Supplemental Data (TAGFAinteractions.xlsx, PLFAinteractions.xlsx, and CarcassInteractions.xlsx) and Figures (Circos Plots). (ZIP 22719 kb) [file 12864_2016_3235_MOESM1_ESM.zip › PL17.png]

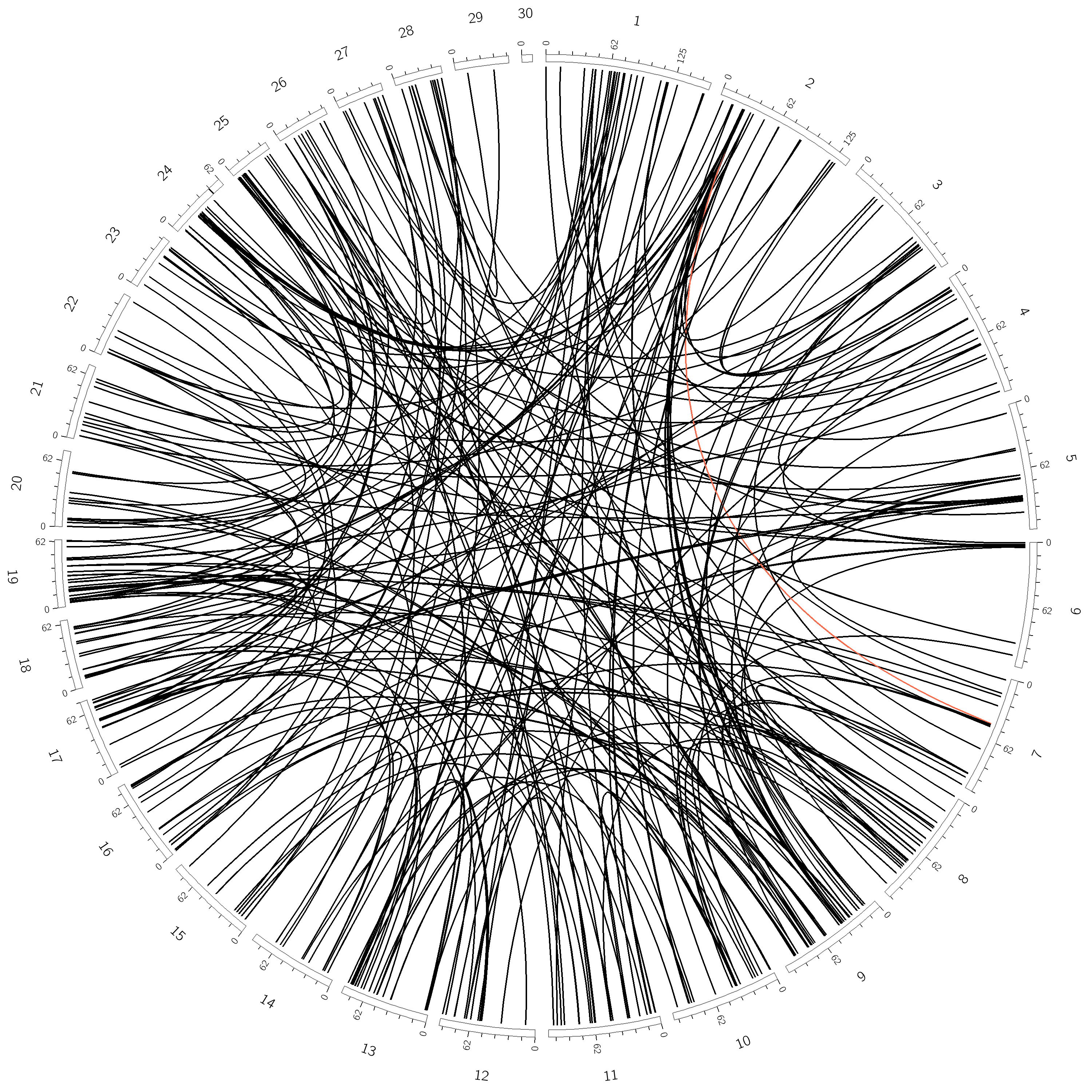

Supplement: Additional file 1: — Supplemental Data (TAGFAinteractions.xlsx, PLFAinteractions.xlsx, and CarcassInteractions.xlsx) and Figures (Circos Plots). (ZIP 22719 kb) [file 12864_2016_3235_MOESM1_ESM.zip › PL171.png]

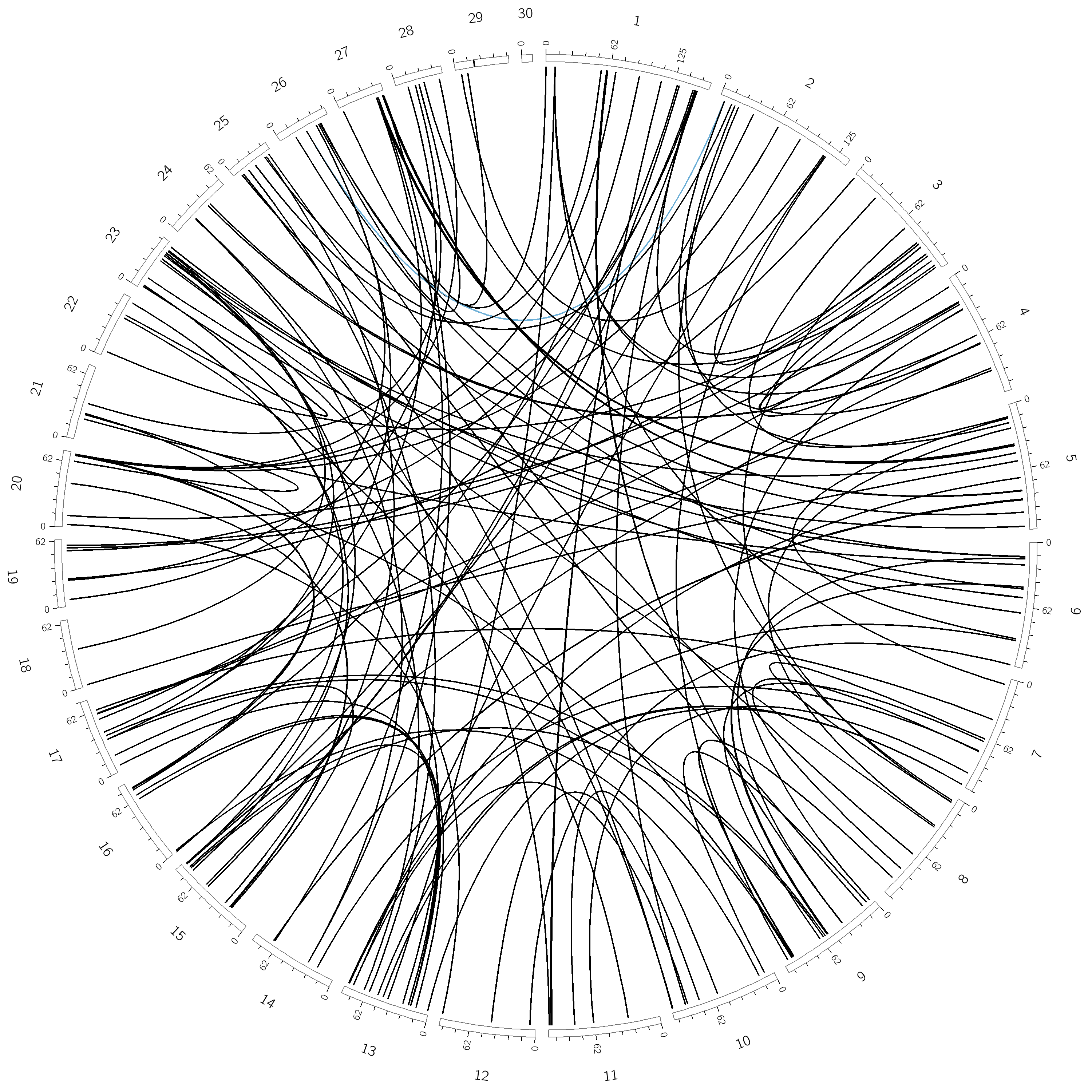

Supplement: Additional file 1: — Supplemental Data (TAGFAinteractions.xlsx, PLFAinteractions.xlsx, and CarcassInteractions.xlsx) and Figures (Circos Plots). (ZIP 22719 kb) [file 12864_2016_3235_MOESM1_ESM.zip › PL18.png]

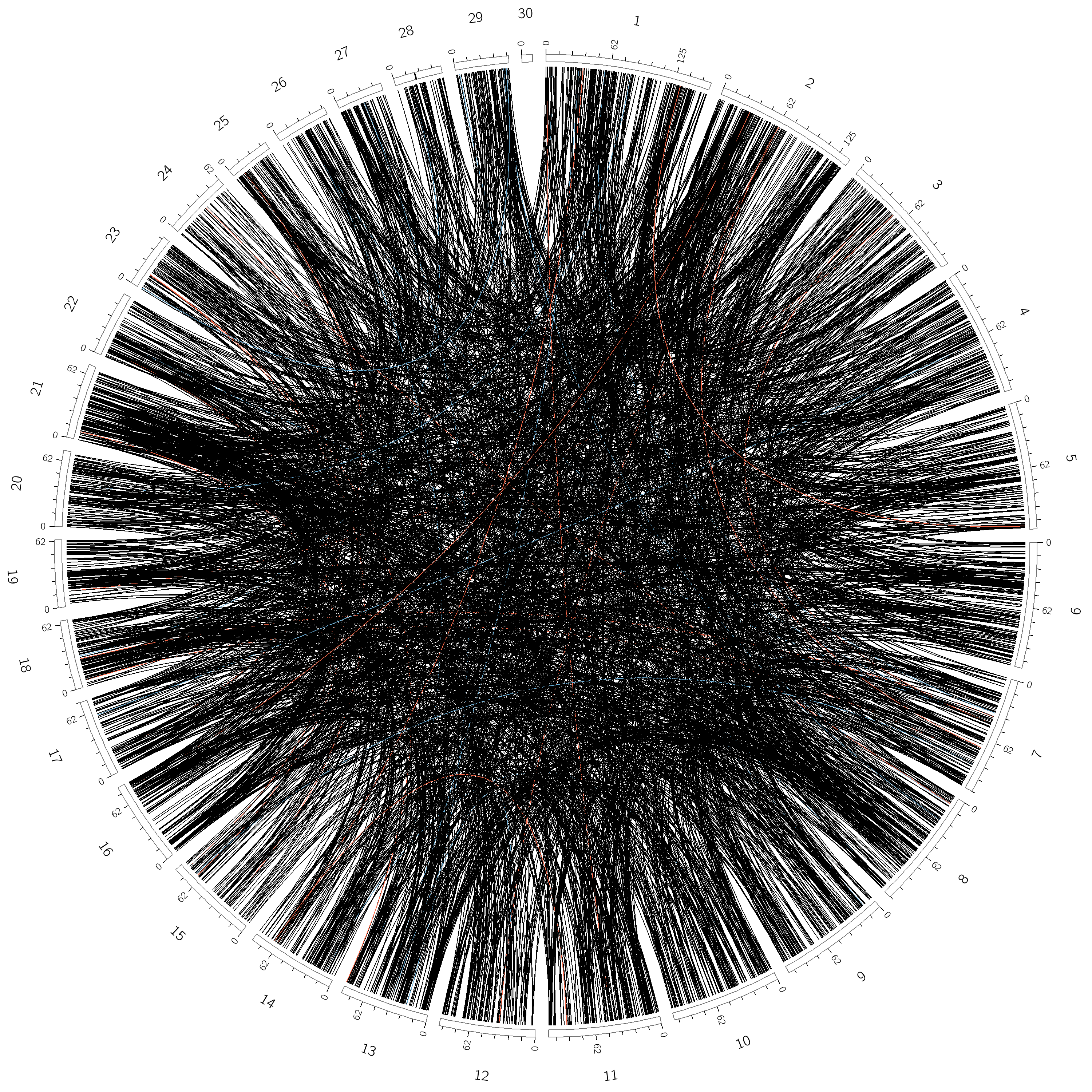

Supplement: Additional file 1: — Supplemental Data (TAGFAinteractions.xlsx, PLFAinteractions.xlsx, and CarcassInteractions.xlsx) and Figures (Circos Plots). (ZIP 22719 kb) [file 12864_2016_3235_MOESM1_ESM.zip › PL181C11.png]

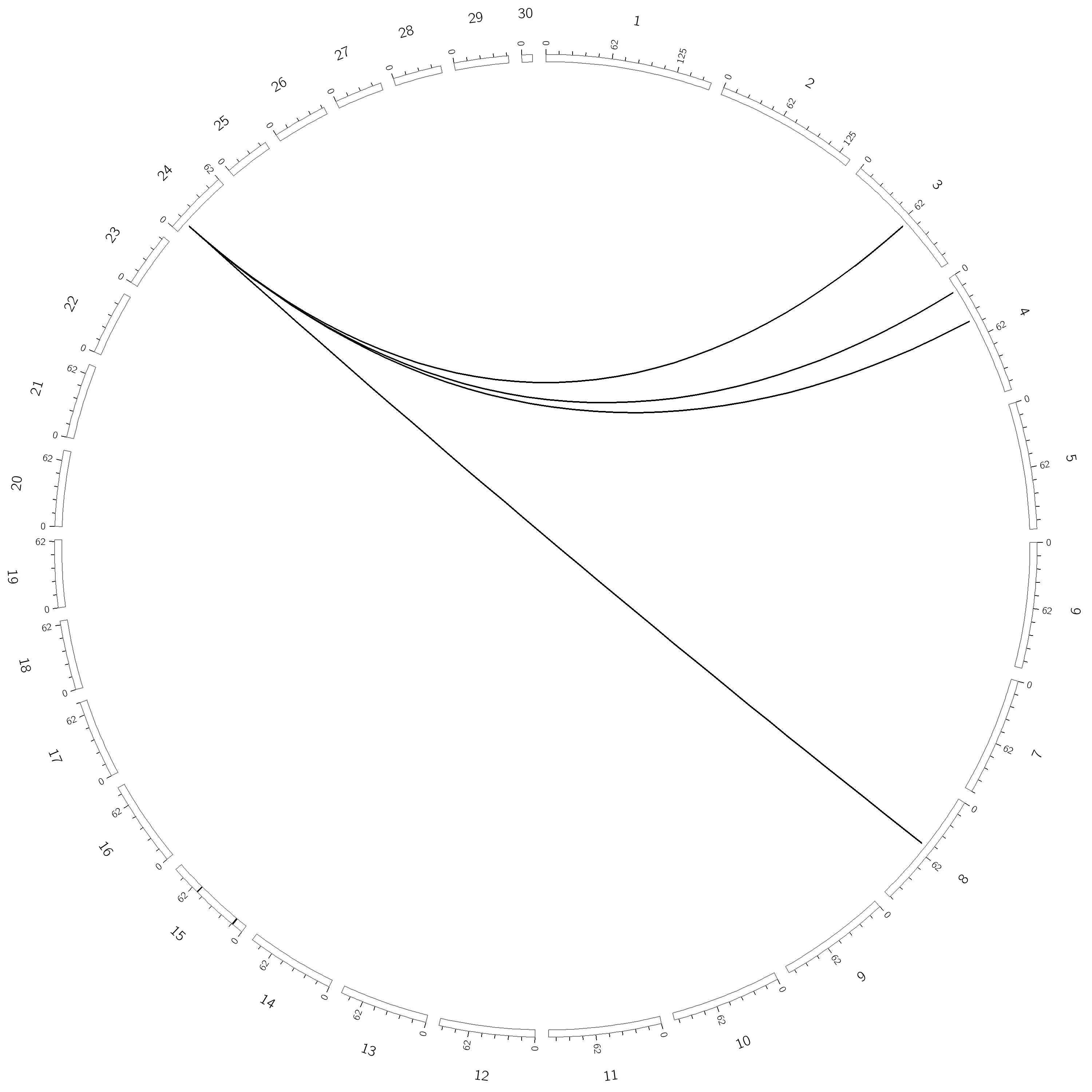

Supplement: Additional file 1: — Supplemental Data (TAGFAinteractions.xlsx, PLFAinteractions.xlsx, and CarcassInteractions.xlsx) and Figures (Circos Plots). (ZIP 22719 kb) [file 12864_2016_3235_MOESM1_ESM.zip › PL181C13.png]

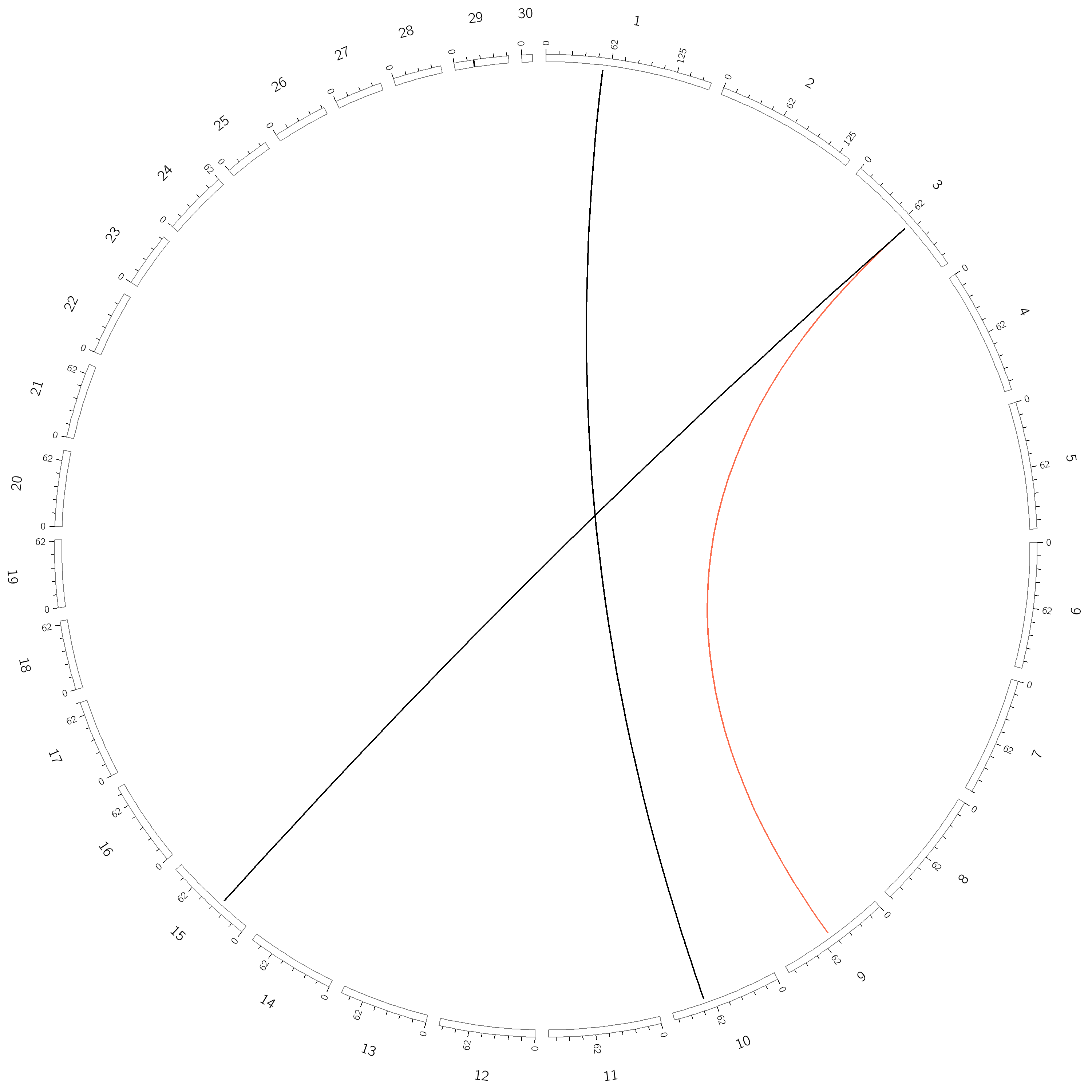

Supplement: Additional file 1: — Supplemental Data (TAGFAinteractions.xlsx, PLFAinteractions.xlsx, and CarcassInteractions.xlsx) and Figures (Circos Plots). (ZIP 22719 kb) [file 12864_2016_3235_MOESM1_ESM.zip › PL22.png]

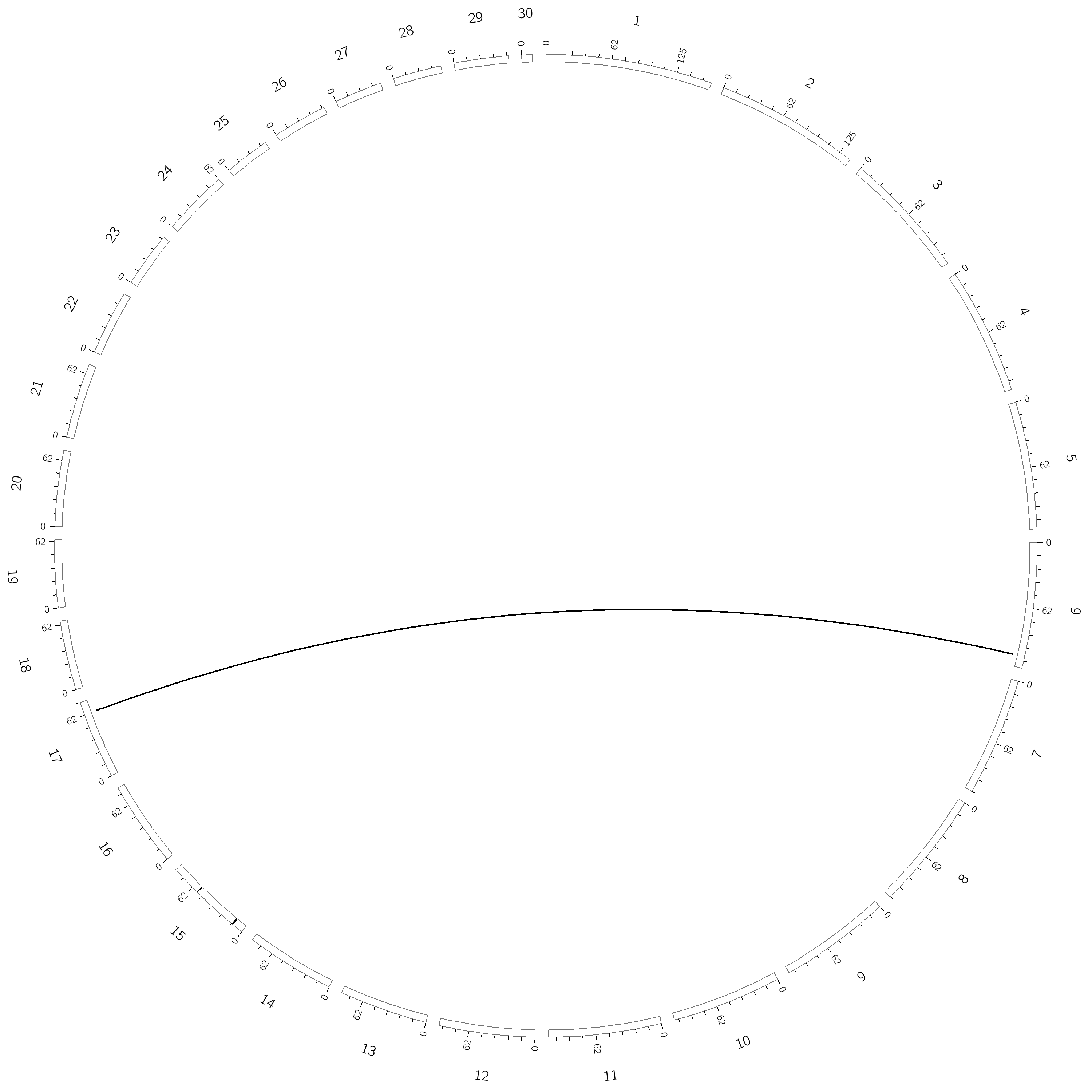

Supplement: Additional file 1: — Supplemental Data (TAGFAinteractions.xlsx, PLFAinteractions.xlsx, and CarcassInteractions.xlsx) and Figures (Circos Plots). (ZIP 22719 kb) [file 12864_2016_3235_MOESM1_ESM.zip › PL226.png]

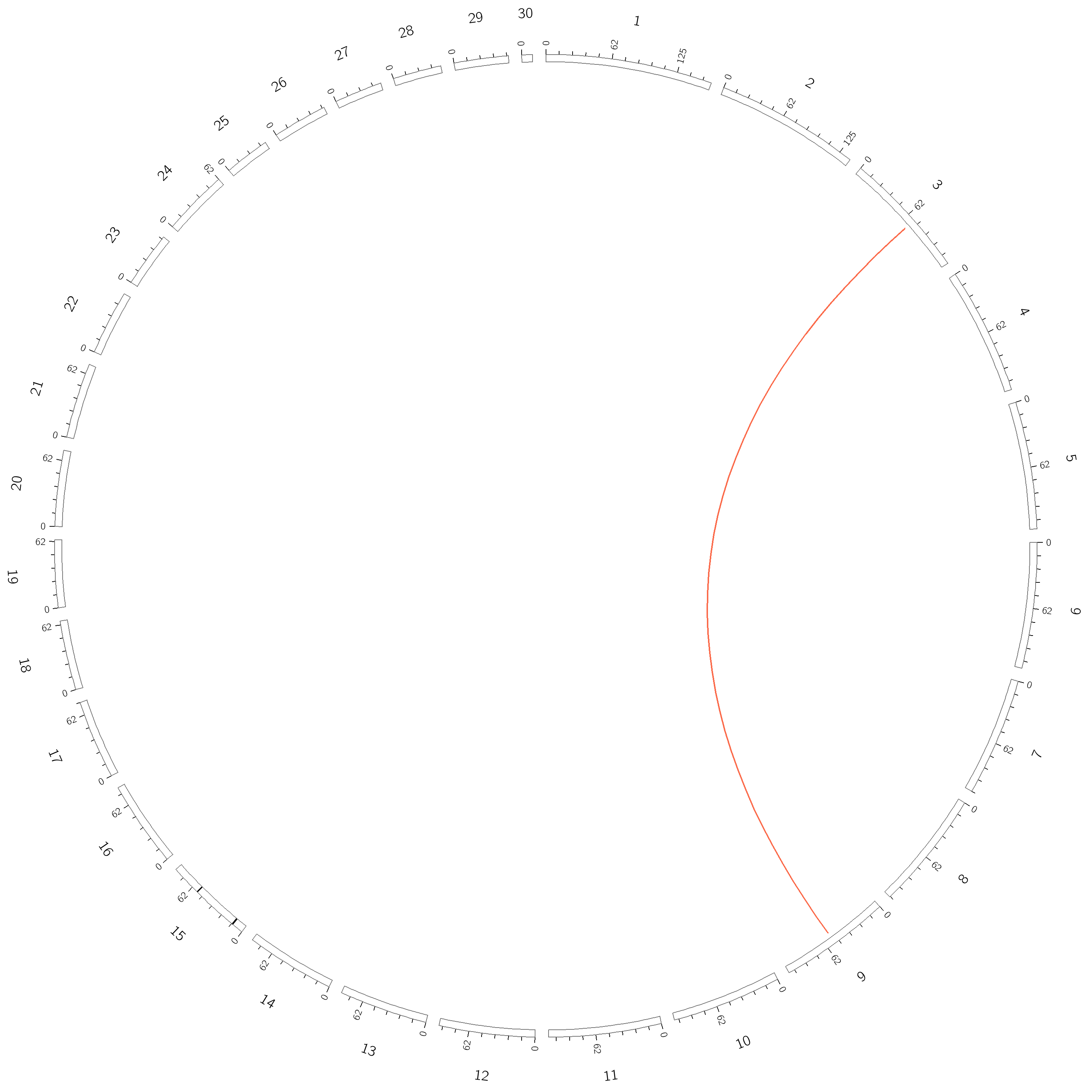

Supplement: Additional file 1: — Supplemental Data (TAGFAinteractions.xlsx, PLFAinteractions.xlsx, and CarcassInteractions.xlsx) and Figures (Circos Plots). (ZIP 22719 kb) [file 12864_2016_3235_MOESM1_ESM.zip › PL23.png]

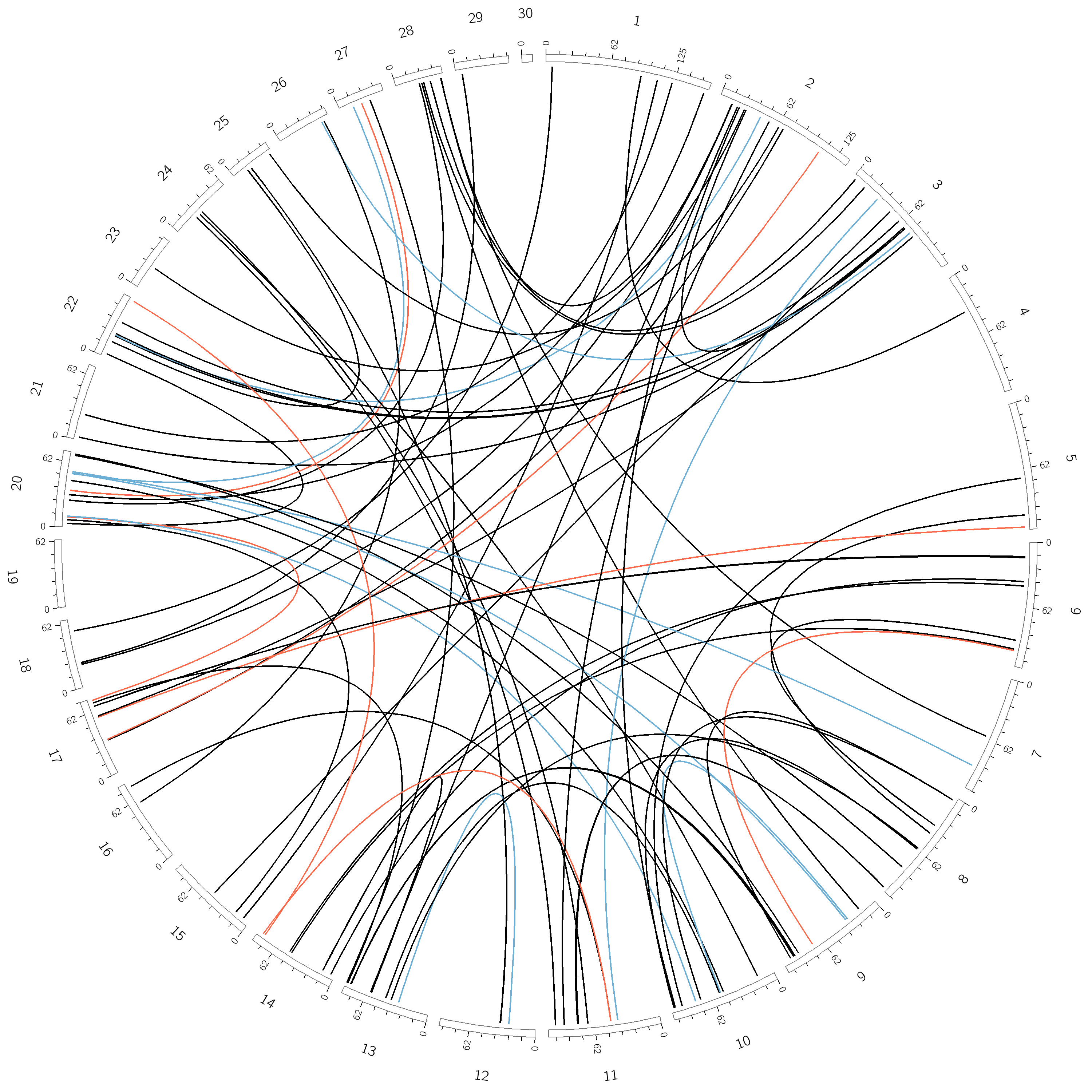

Supplement: Additional file 1: — Supplemental Data (TAGFAinteractions.xlsx, PLFAinteractions.xlsx, and CarcassInteractions.xlsx) and Figures (Circos Plots). (ZIP 22719 kb) [file 12864_2016_3235_MOESM1_ESM.zip › PLCLAC12.png]

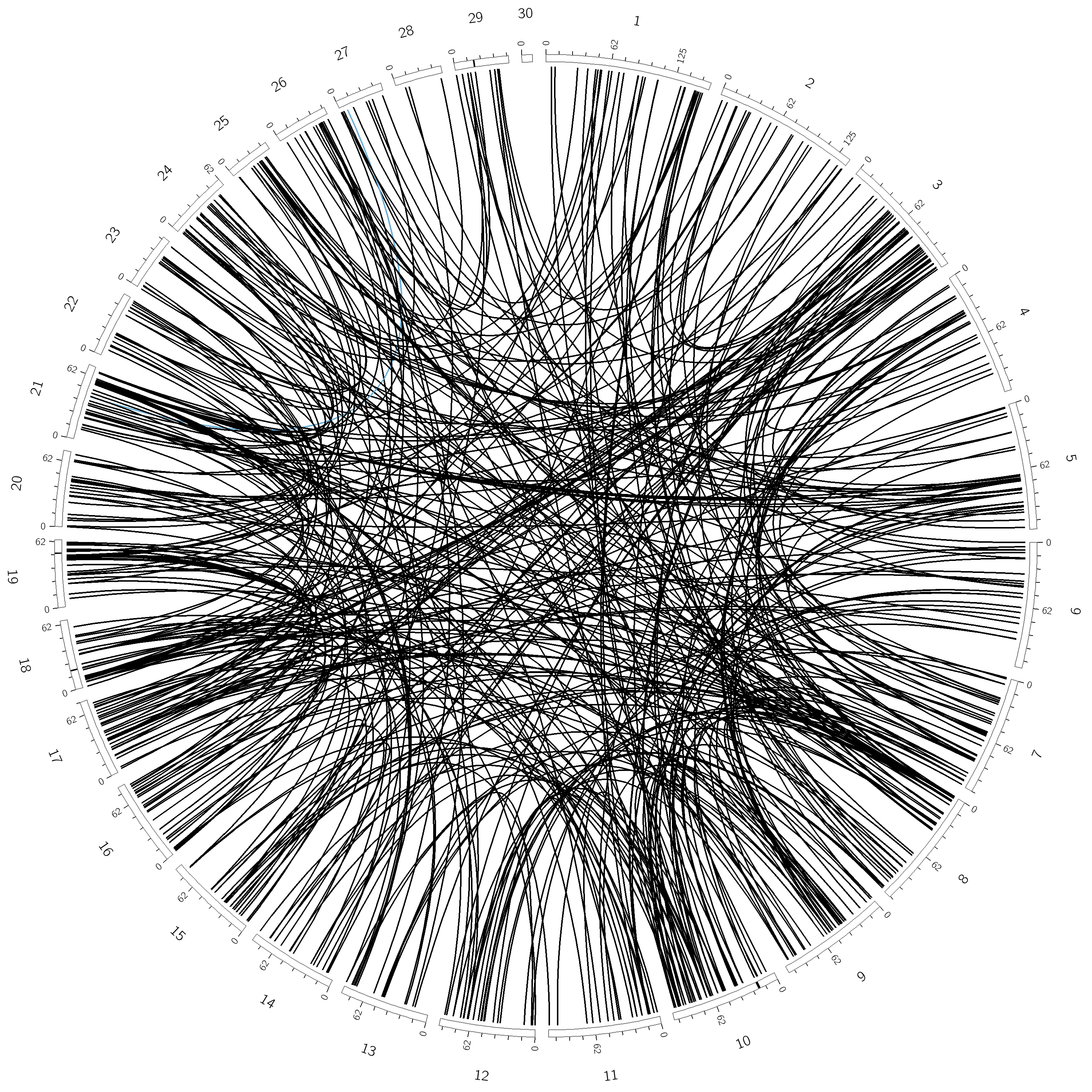

Supplement: Additional file 1: — Supplemental Data (TAGFAinteractions.xlsx, PLFAinteractions.xlsx, and CarcassInteractions.xlsx) and Figures (Circos Plots). (ZIP 22719 kb) [file 12864_2016_3235_MOESM1_ESM.zip › PLLCFA.png]

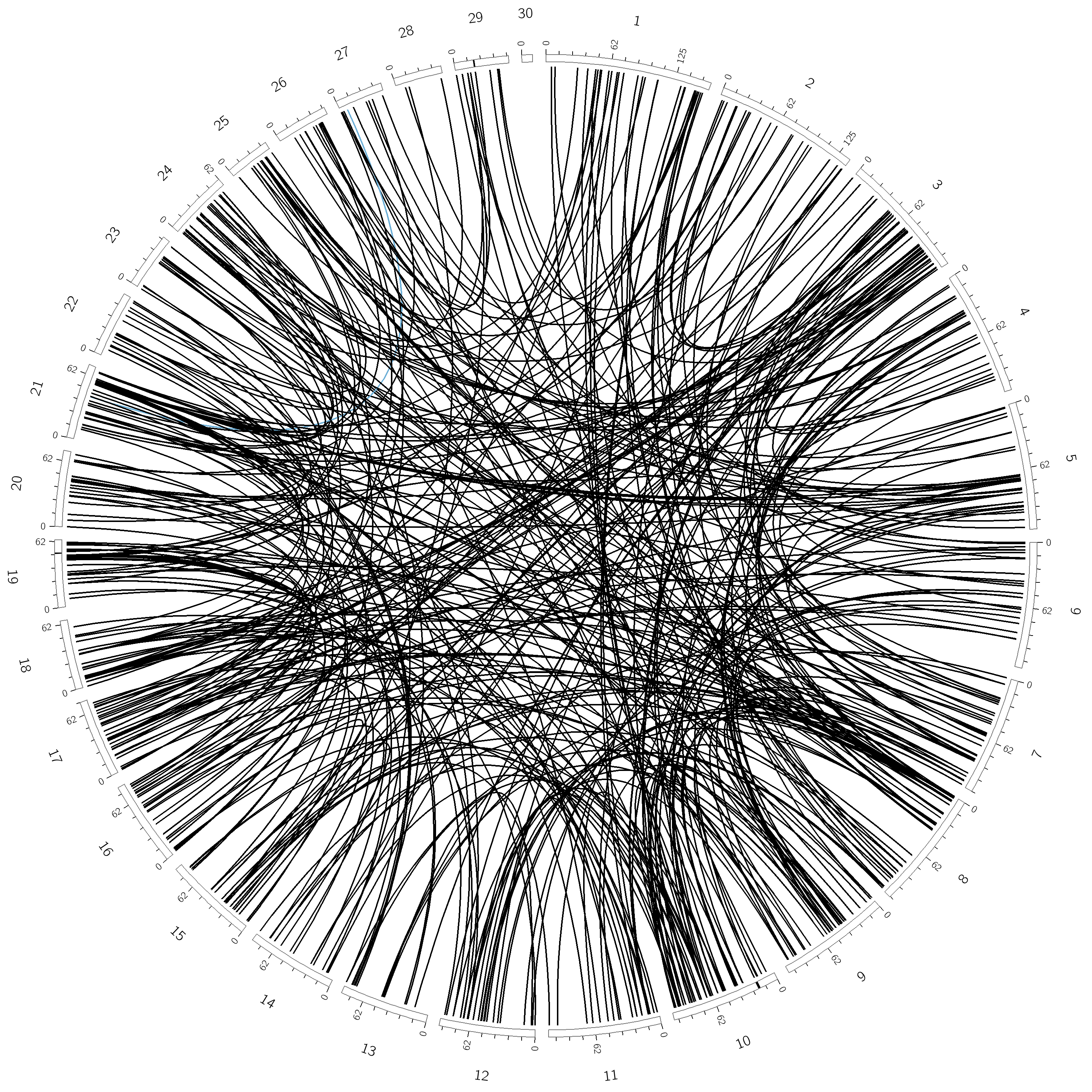

Supplement: Additional file 1: — Supplemental Data (TAGFAinteractions.xlsx, PLFAinteractions.xlsx, and CarcassInteractions.xlsx) and Figures (Circos Plots). (ZIP 22719 kb) [file 12864_2016_3235_MOESM1_ESM.zip › PLMCFA.png]

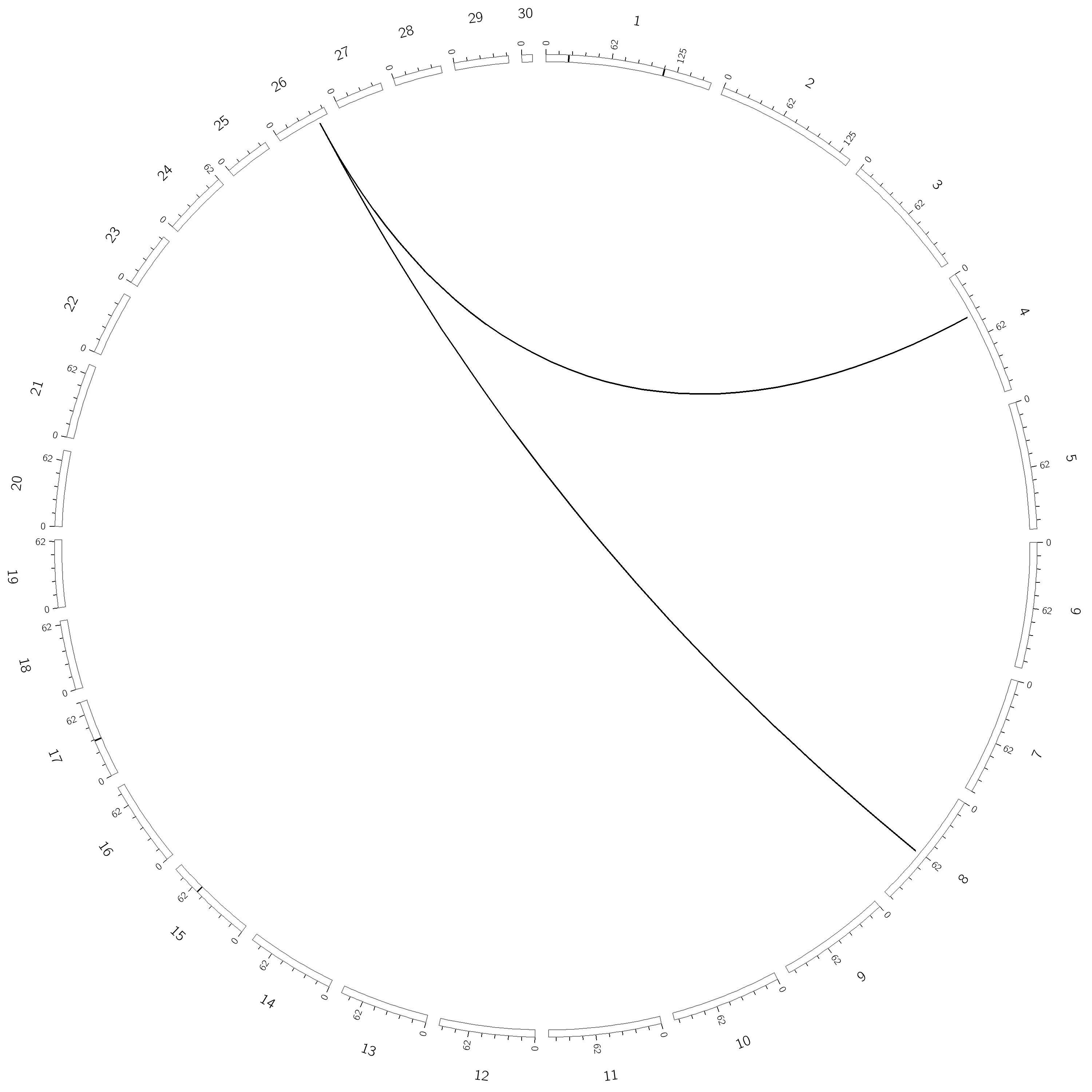

Supplement: Additional file 1: — Supplemental Data (TAGFAinteractions.xlsx, PLFAinteractions.xlsx, and CarcassInteractions.xlsx) and Figures (Circos Plots). (ZIP 22719 kb) [file 12864_2016_3235_MOESM1_ESM.zip › PLn3n6.png]

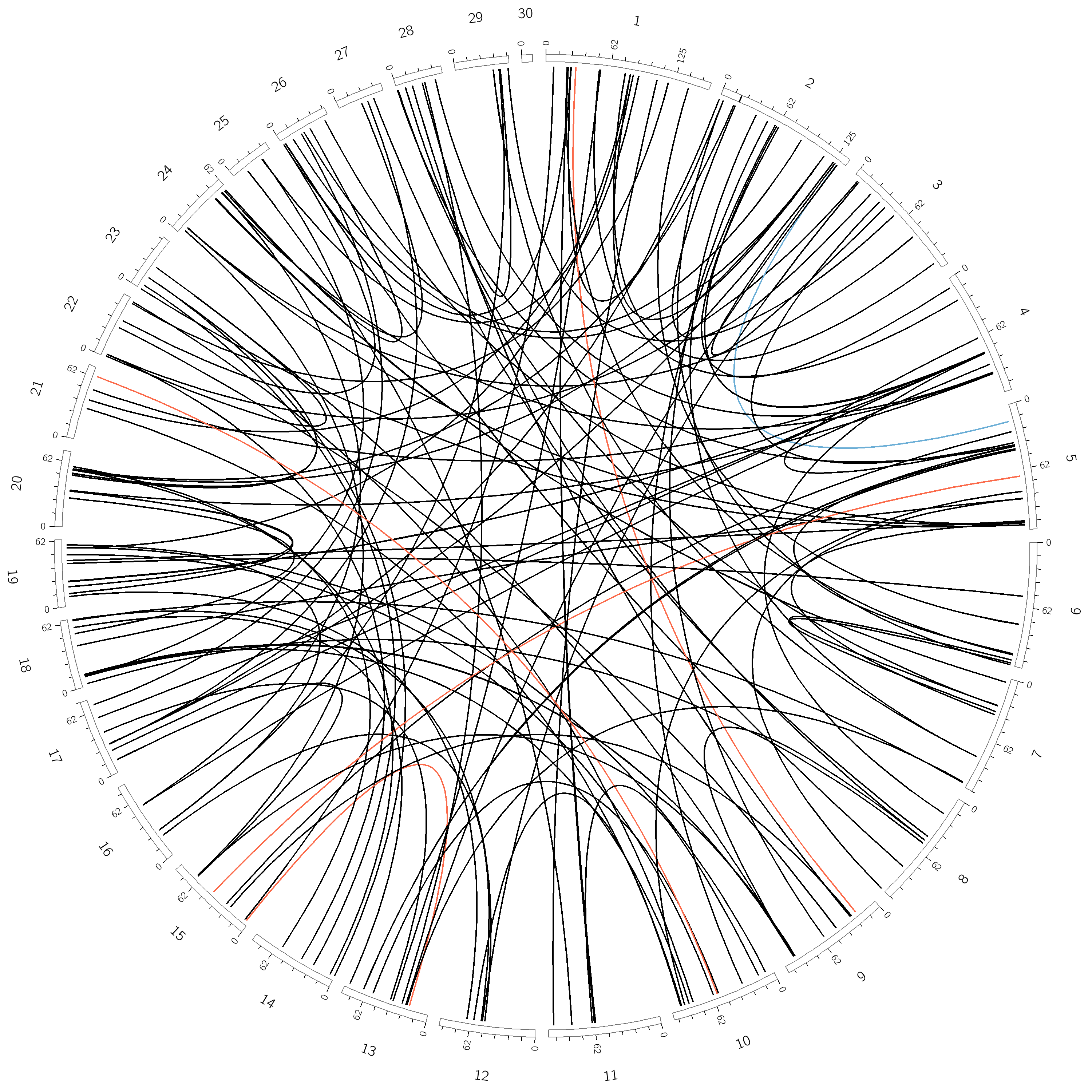

Supplement: Additional file 1: — Supplemental Data (TAGFAinteractions.xlsx, PLFAinteractions.xlsx, and CarcassInteractions.xlsx) and Figures (Circos Plots). (ZIP 22719 kb) [file 12864_2016_3235_MOESM1_ESM.zip › PLn6.png]

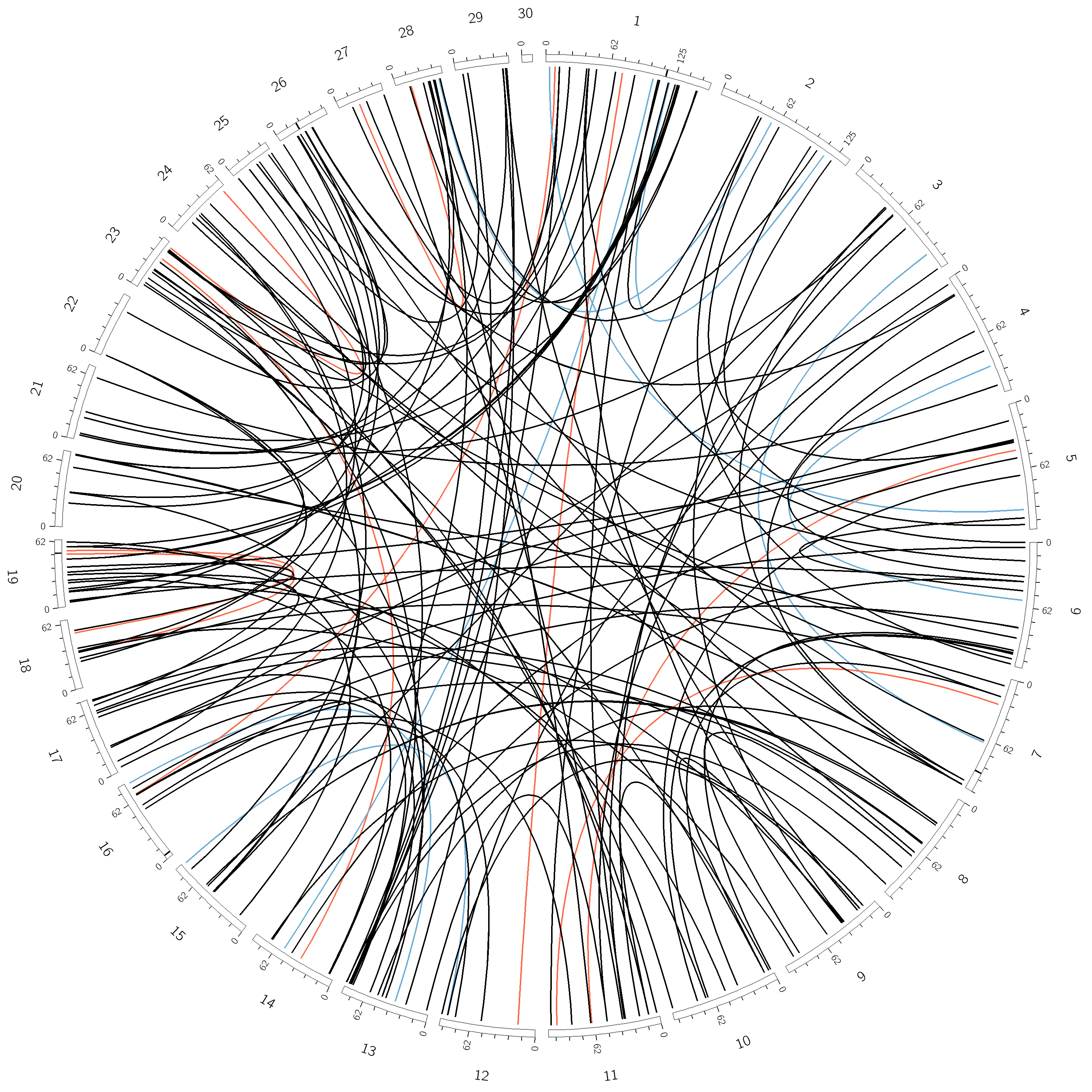

Supplement: Additional file 1: — Supplemental Data (TAGFAinteractions.xlsx, PLFAinteractions.xlsx, and CarcassInteractions.xlsx) and Figures (Circos Plots). (ZIP 22719 kb) [file 12864_2016_3235_MOESM1_ESM.zip › PLSFA.png]

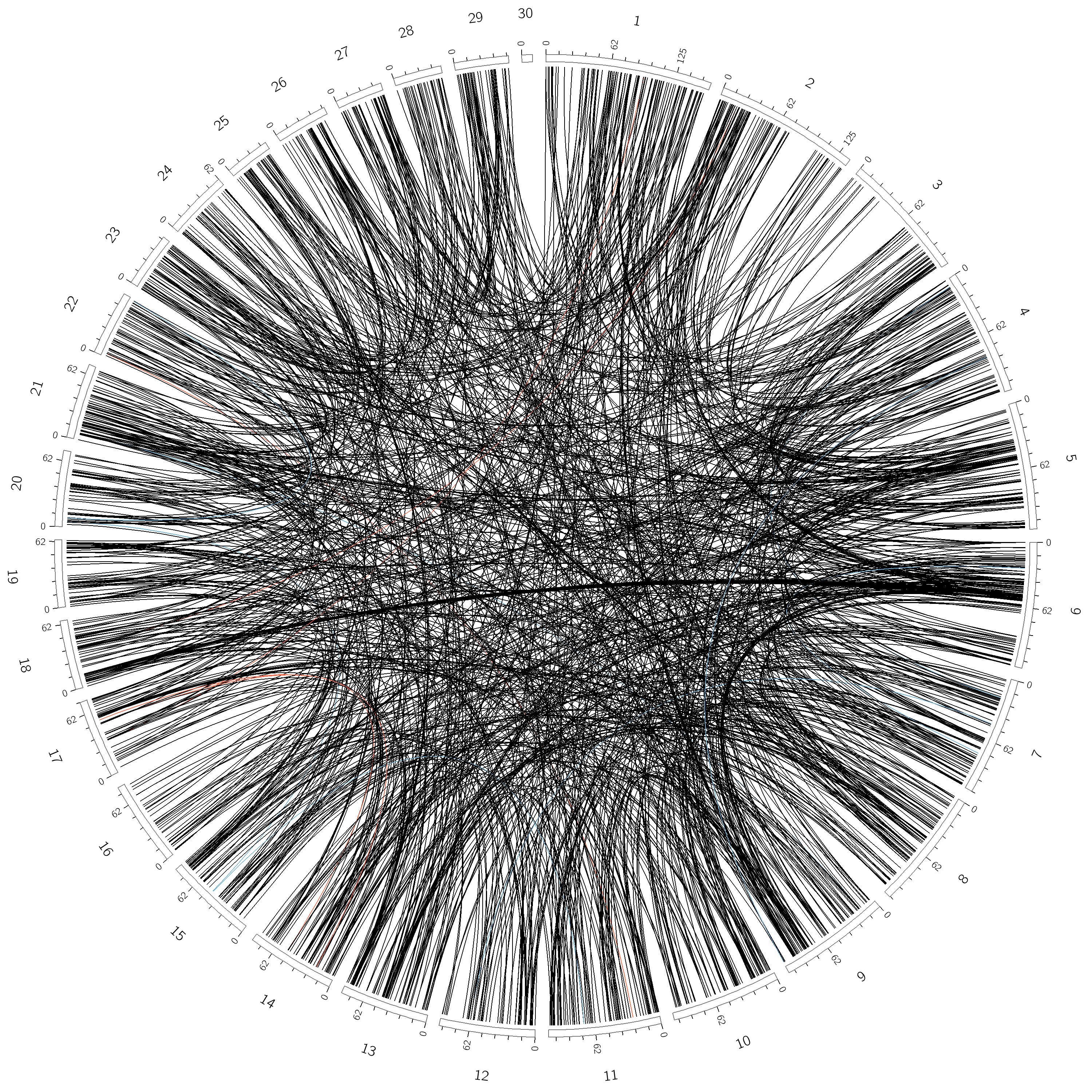

Supplement: Additional file 1: — Supplemental Data (TAGFAinteractions.xlsx, PLFAinteractions.xlsx, and CarcassInteractions.xlsx) and Figures (Circos Plots). (ZIP 22719 kb) [file 12864_2016_3235_MOESM1_ESM.zip › PLT10T11.png]

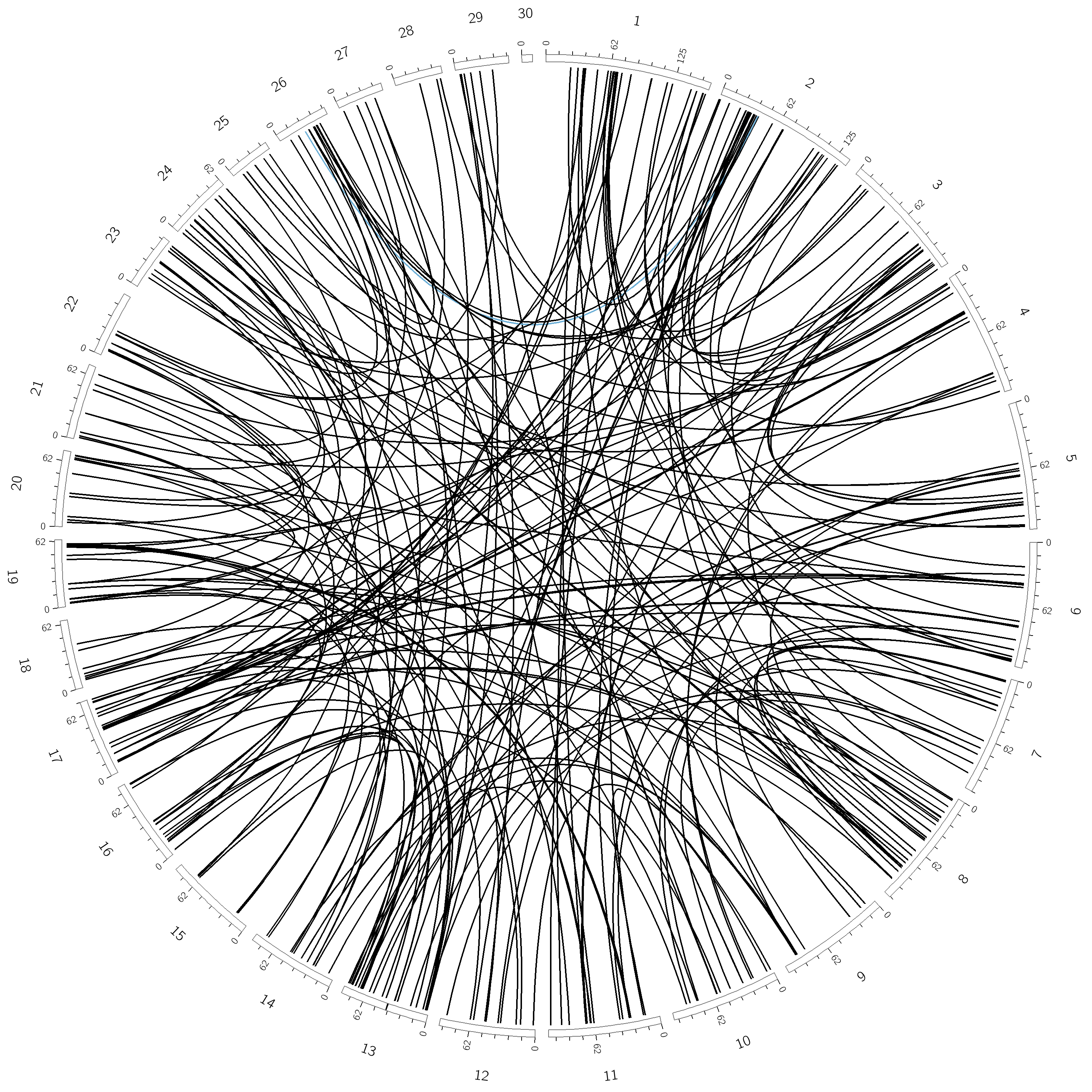

Supplement: Additional file 1: — Supplemental Data (TAGFAinteractions.xlsx, PLFAinteractions.xlsx, and CarcassInteractions.xlsx) and Figures (Circos Plots). (ZIP 22719 kb) [file 12864_2016_3235_MOESM1_ESM.zip › PLT15.png]

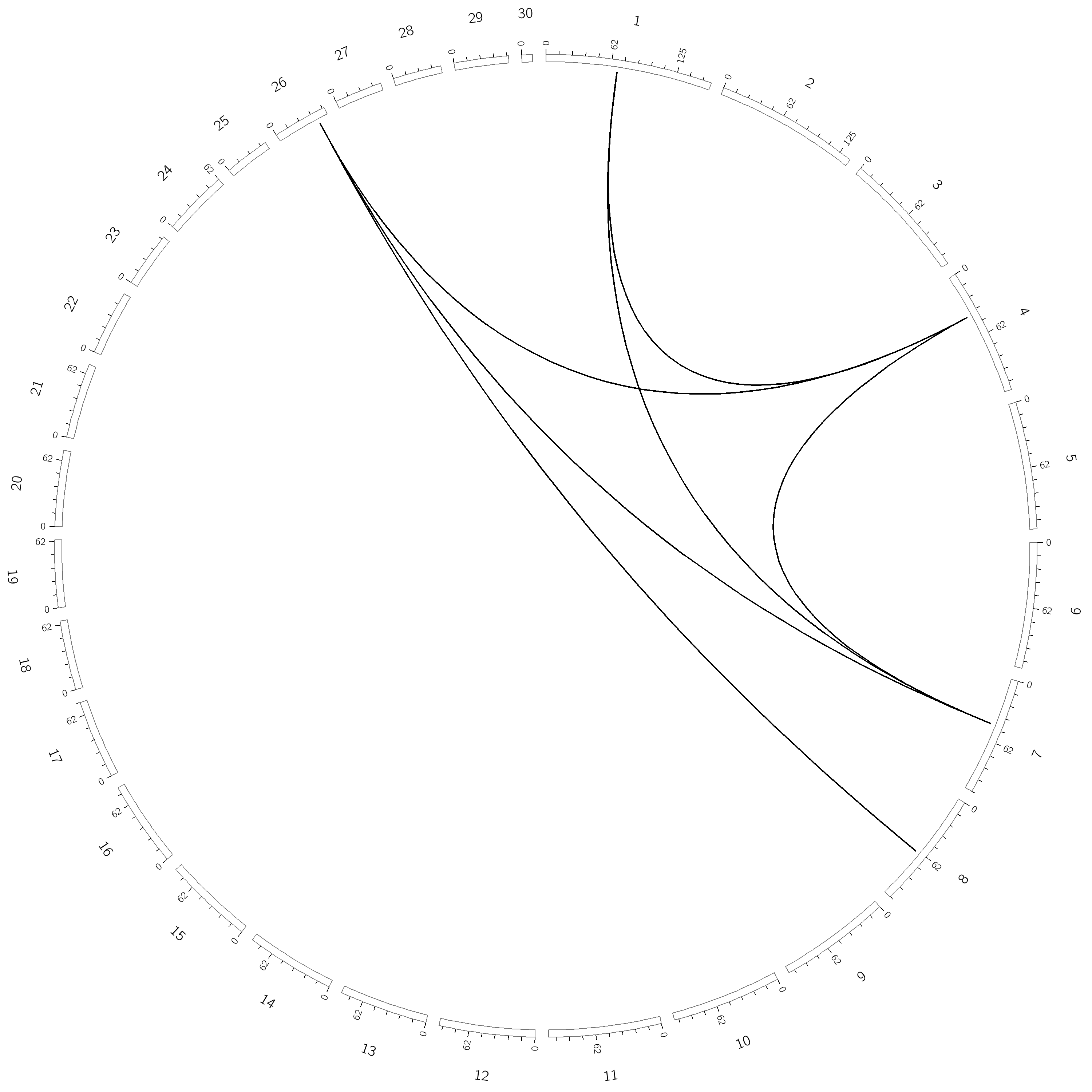

Supplement: Additional file 1: — Supplemental Data (TAGFAinteractions.xlsx, PLFAinteractions.xlsx, and CarcassInteractions.xlsx) and Figures (Circos Plots). (ZIP 22719 kb) [file 12864_2016_3235_MOESM1_ESM.zip › TAG12.png]

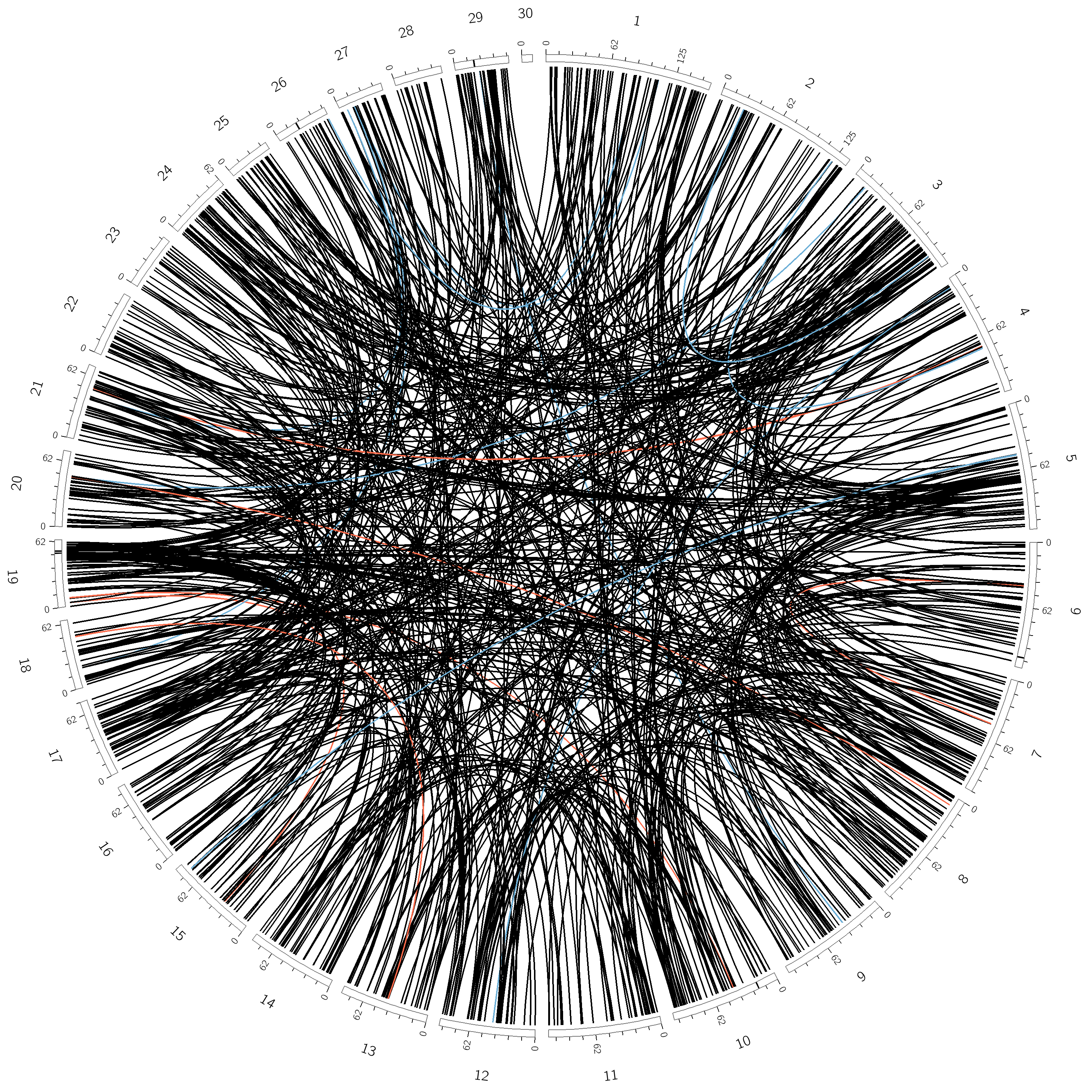

Supplement: Additional file 1: — Supplemental Data (TAGFAinteractions.xlsx, PLFAinteractions.xlsx, and CarcassInteractions.xlsx) and Figures (Circos Plots). (ZIP 22719 kb) [file 12864_2016_3235_MOESM1_ESM.zip › TAG14.png]

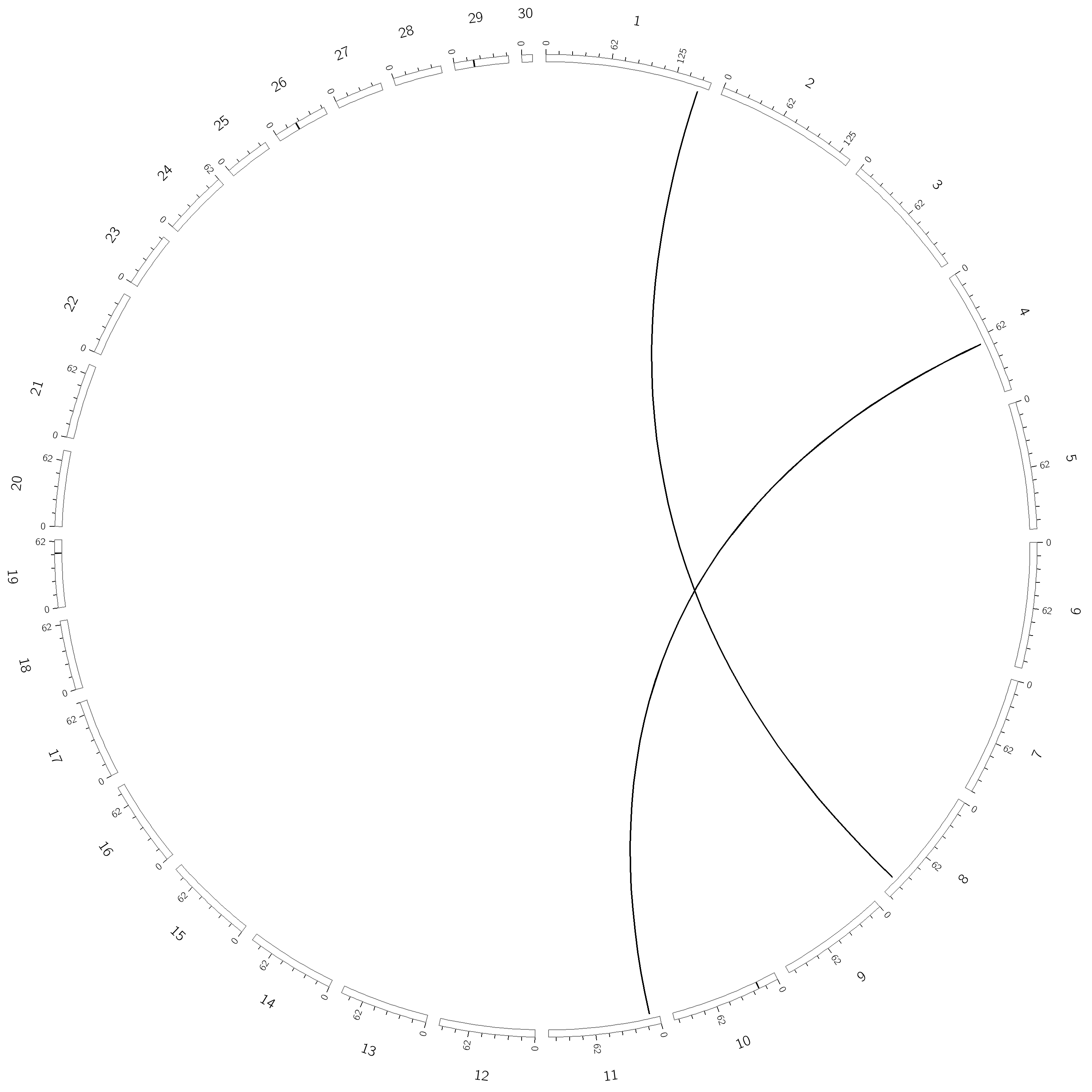

Supplement: Additional file 1: — Supplemental Data (TAGFAinteractions.xlsx, PLFAinteractions.xlsx, and CarcassInteractions.xlsx) and Figures (Circos Plots). (ZIP 22719 kb) [file 12864_2016_3235_MOESM1_ESM.zip › TAG141.png]

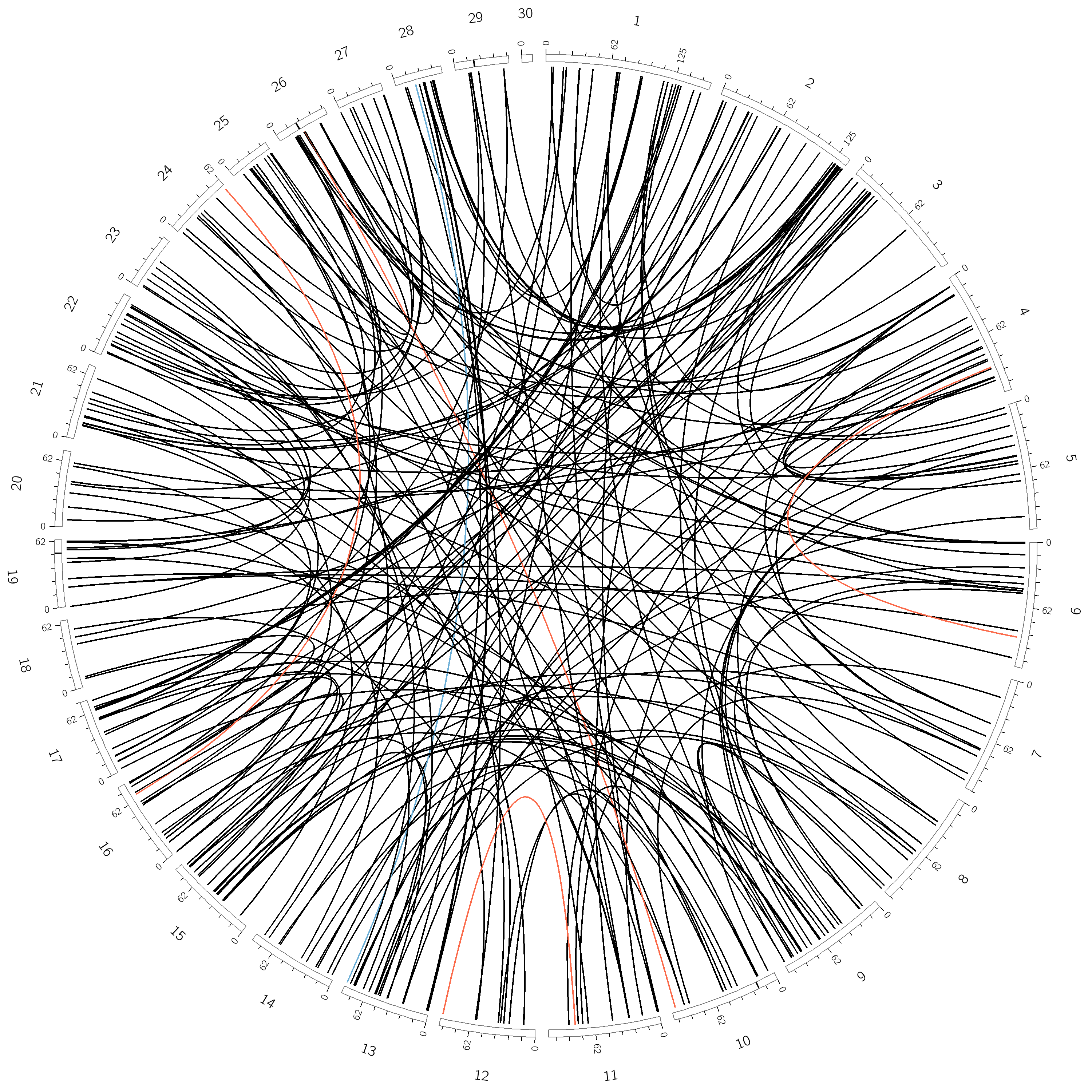

Supplement: Additional file 1: — Supplemental Data (TAGFAinteractions.xlsx, PLFAinteractions.xlsx, and CarcassInteractions.xlsx) and Figures (Circos Plots). (ZIP 22719 kb) [file 12864_2016_3235_MOESM1_ESM.zip › TAG161.png]

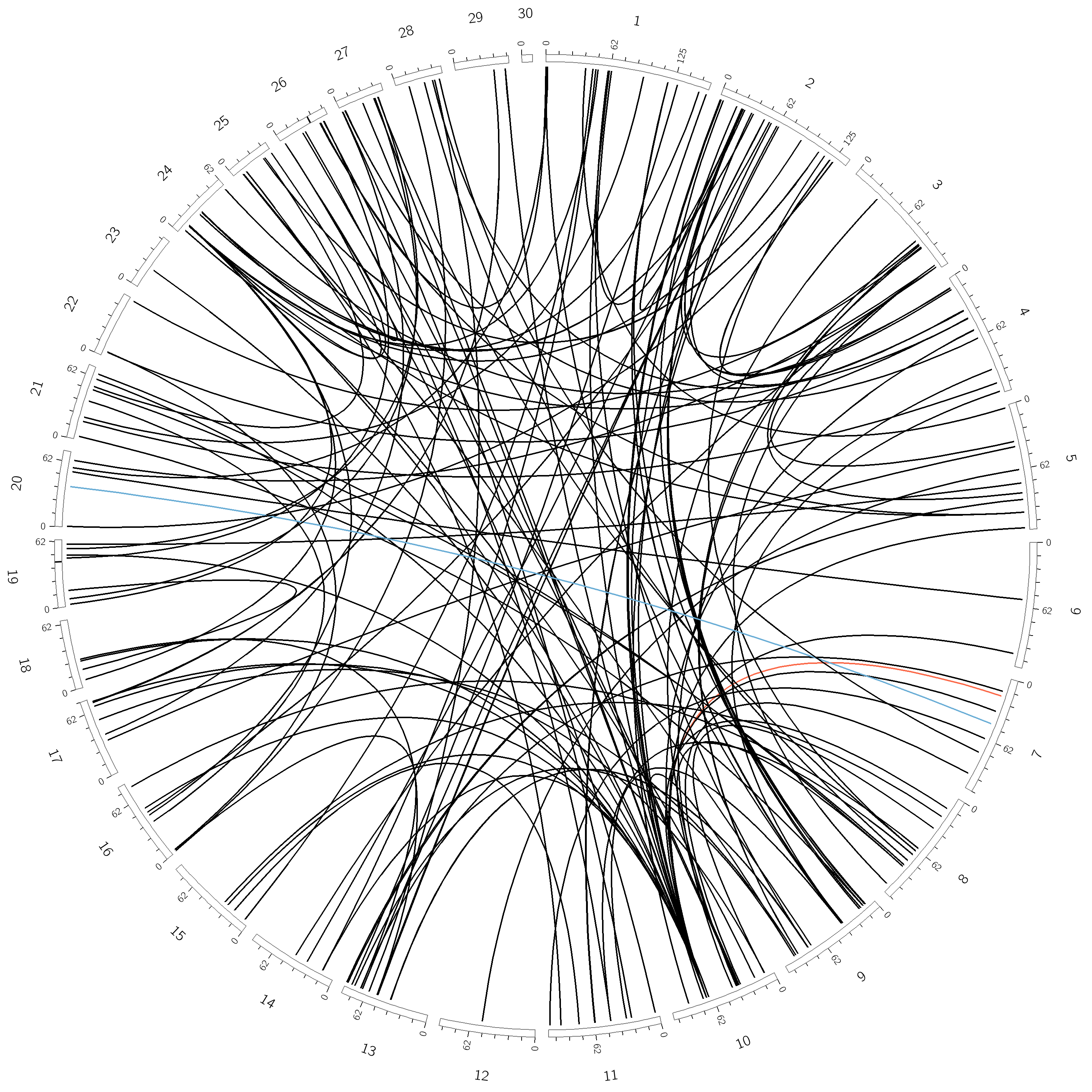

Supplement: Additional file 1: — Supplemental Data (TAGFAinteractions.xlsx, PLFAinteractions.xlsx, and CarcassInteractions.xlsx) and Figures (Circos Plots). (ZIP 22719 kb) [file 12864_2016_3235_MOESM1_ESM.zip › TAG17.png]

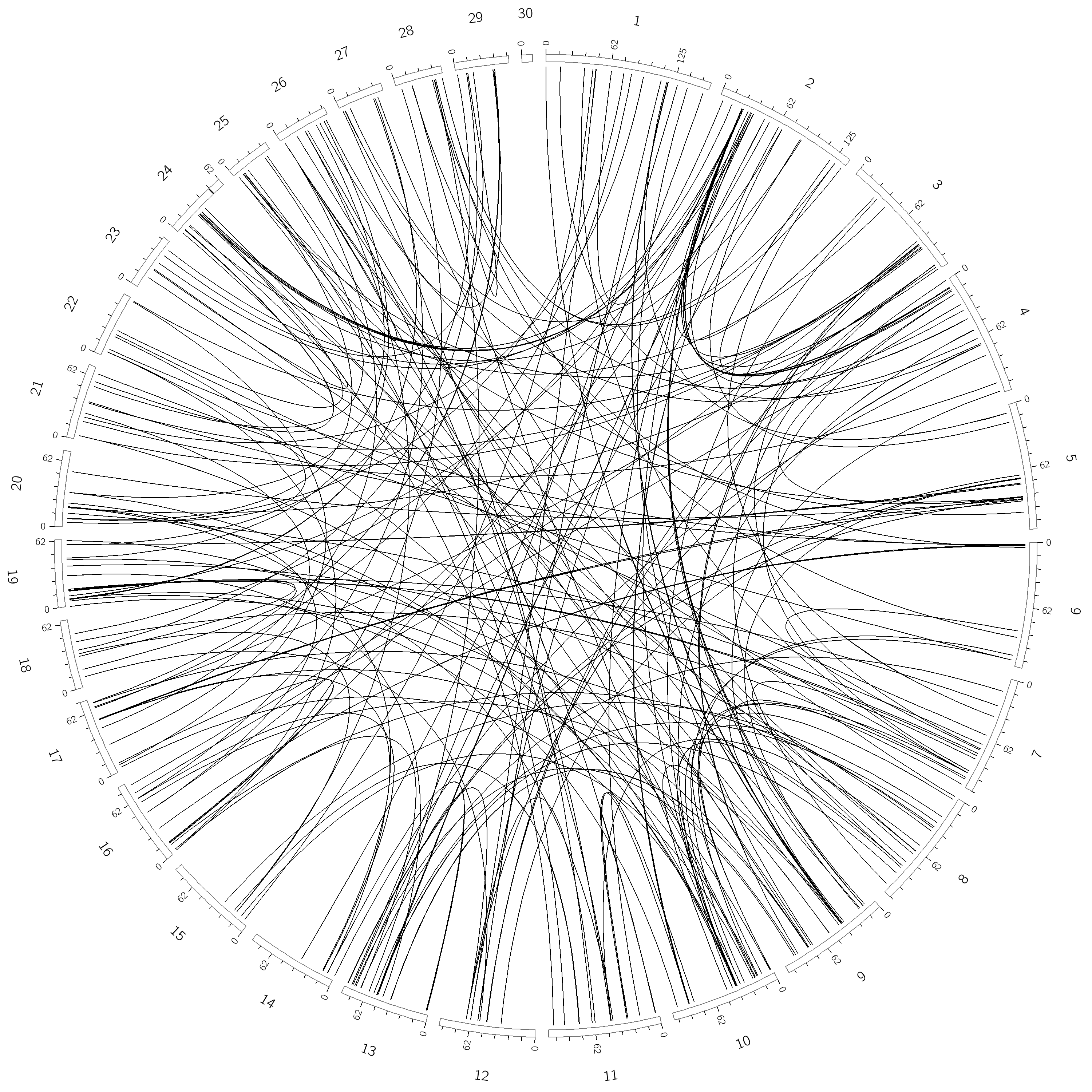

Supplement: Additional file 1: — Supplemental Data (TAGFAinteractions.xlsx, PLFAinteractions.xlsx, and CarcassInteractions.xlsx) and Figures (Circos Plots). (ZIP 22719 kb) [file 12864_2016_3235_MOESM1_ESM.zip › TAG171.png]

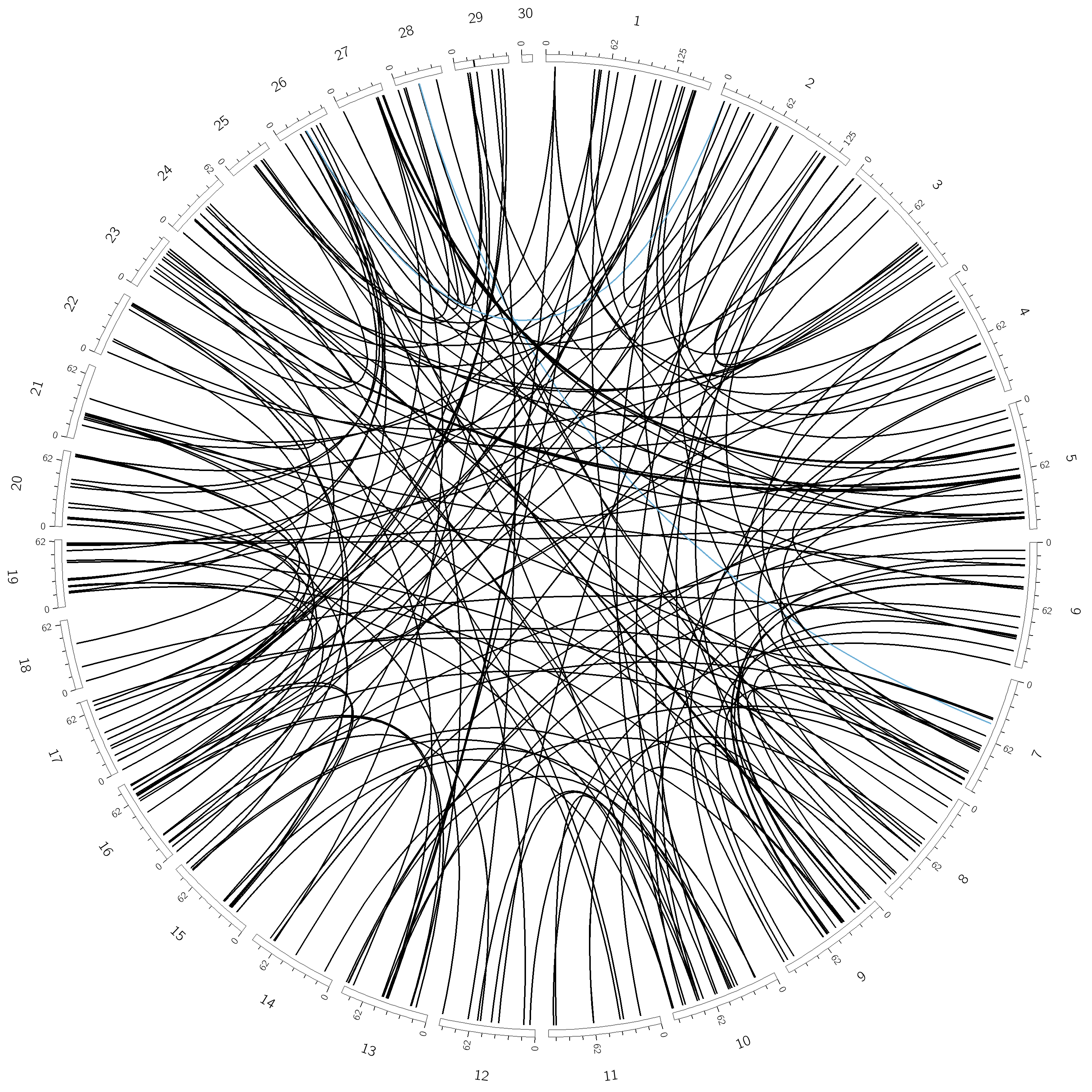

Supplement: Additional file 1: — Supplemental Data (TAGFAinteractions.xlsx, PLFAinteractions.xlsx, and CarcassInteractions.xlsx) and Figures (Circos Plots). (ZIP 22719 kb) [file 12864_2016_3235_MOESM1_ESM.zip › TAG18.png]

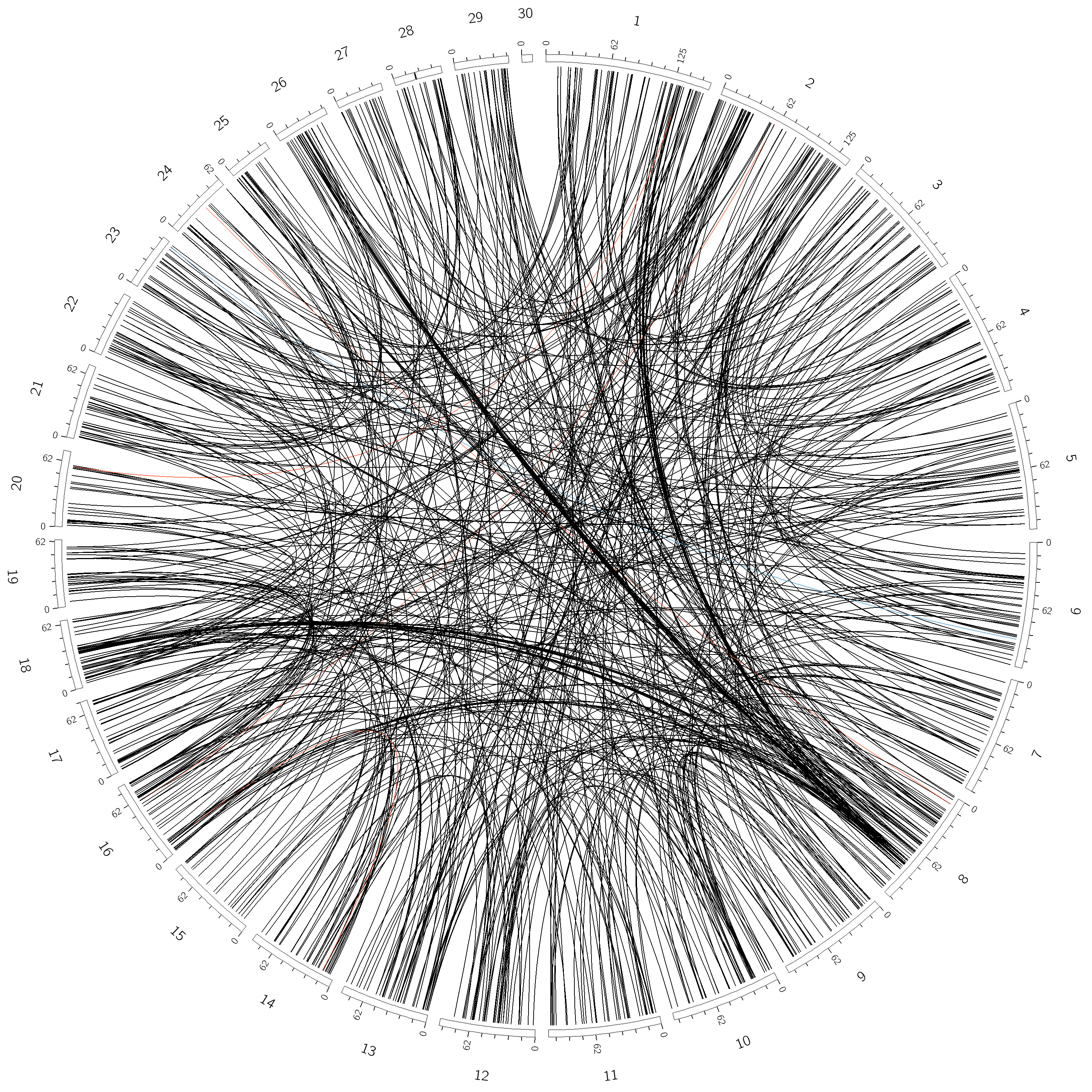

Supplement: Additional file 1: — Supplemental Data (TAGFAinteractions.xlsx, PLFAinteractions.xlsx, and CarcassInteractions.xlsx) and Figures (Circos Plots). (ZIP 22719 kb) [file 12864_2016_3235_MOESM1_ESM.zip › TAG181C11.png]

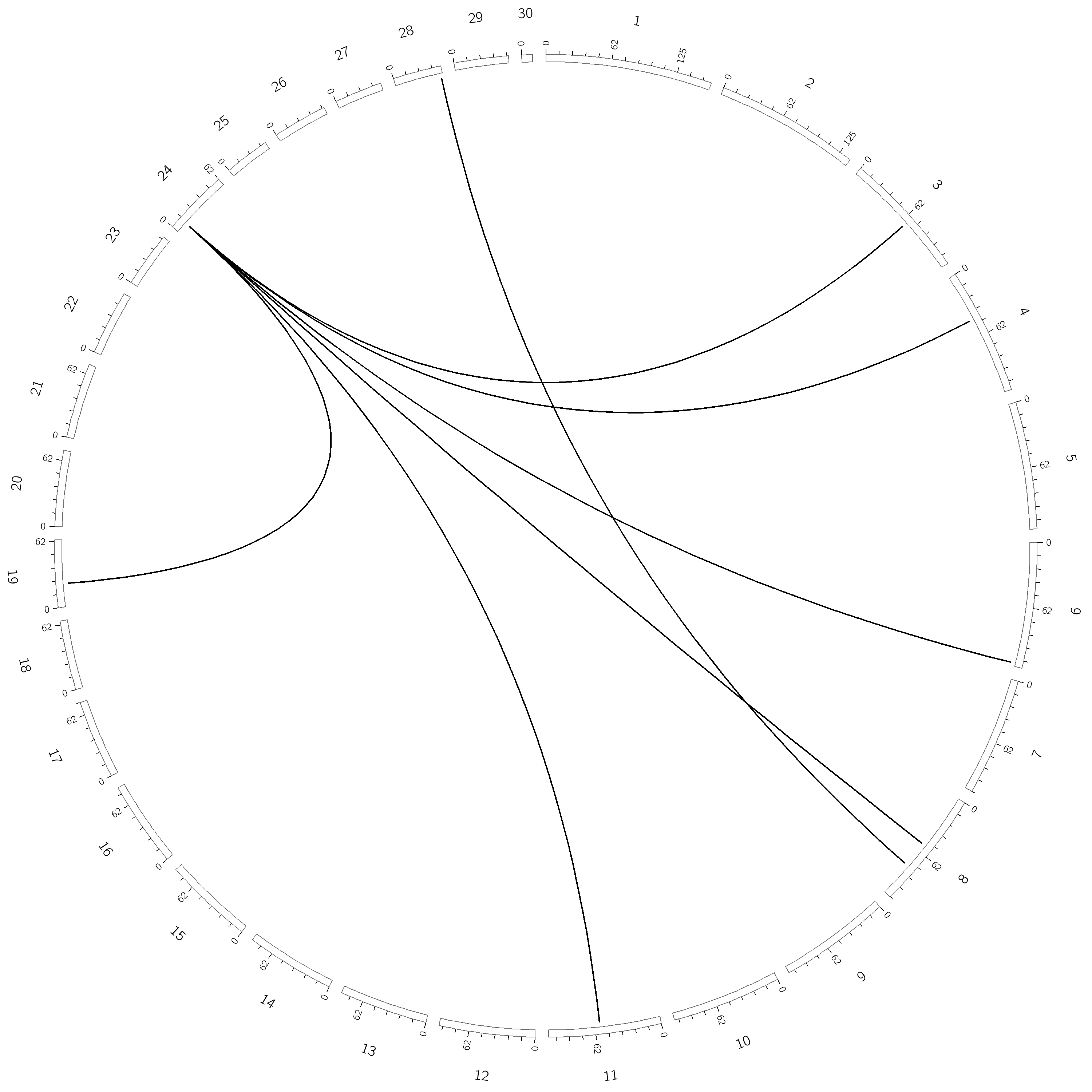

Supplement: Additional file 1: — Supplemental Data (TAGFAinteractions.xlsx, PLFAinteractions.xlsx, and CarcassInteractions.xlsx) and Figures (Circos Plots). (ZIP 22719 kb) [file 12864_2016_3235_MOESM1_ESM.zip › TAG181C13.png]

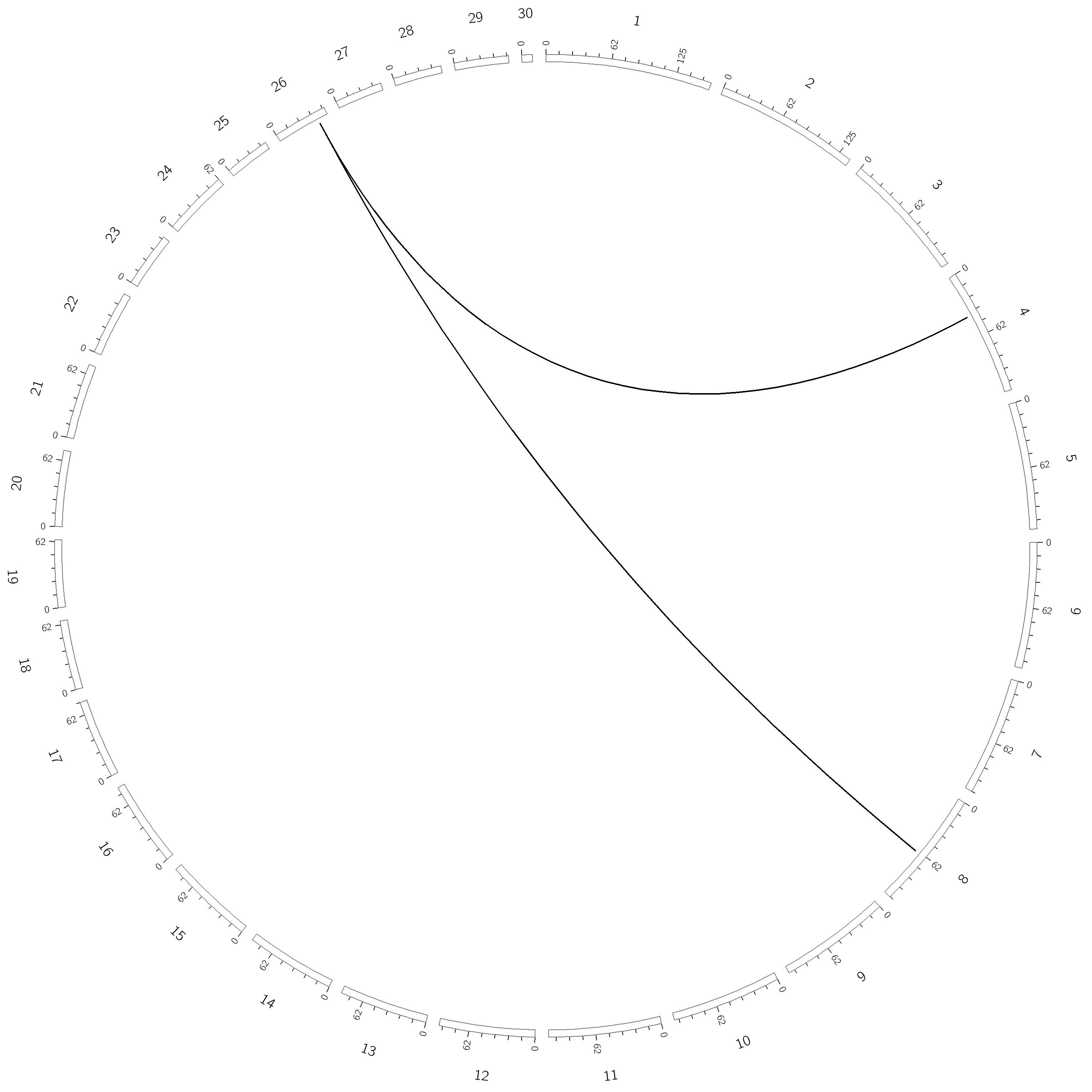

Supplement: Additional file 1: — Supplemental Data (TAGFAinteractions.xlsx, PLFAinteractions.xlsx, and CarcassInteractions.xlsx) and Figures (Circos Plots). (ZIP 22719 kb) [file 12864_2016_3235_MOESM1_ESM.zip › TAG202.png]

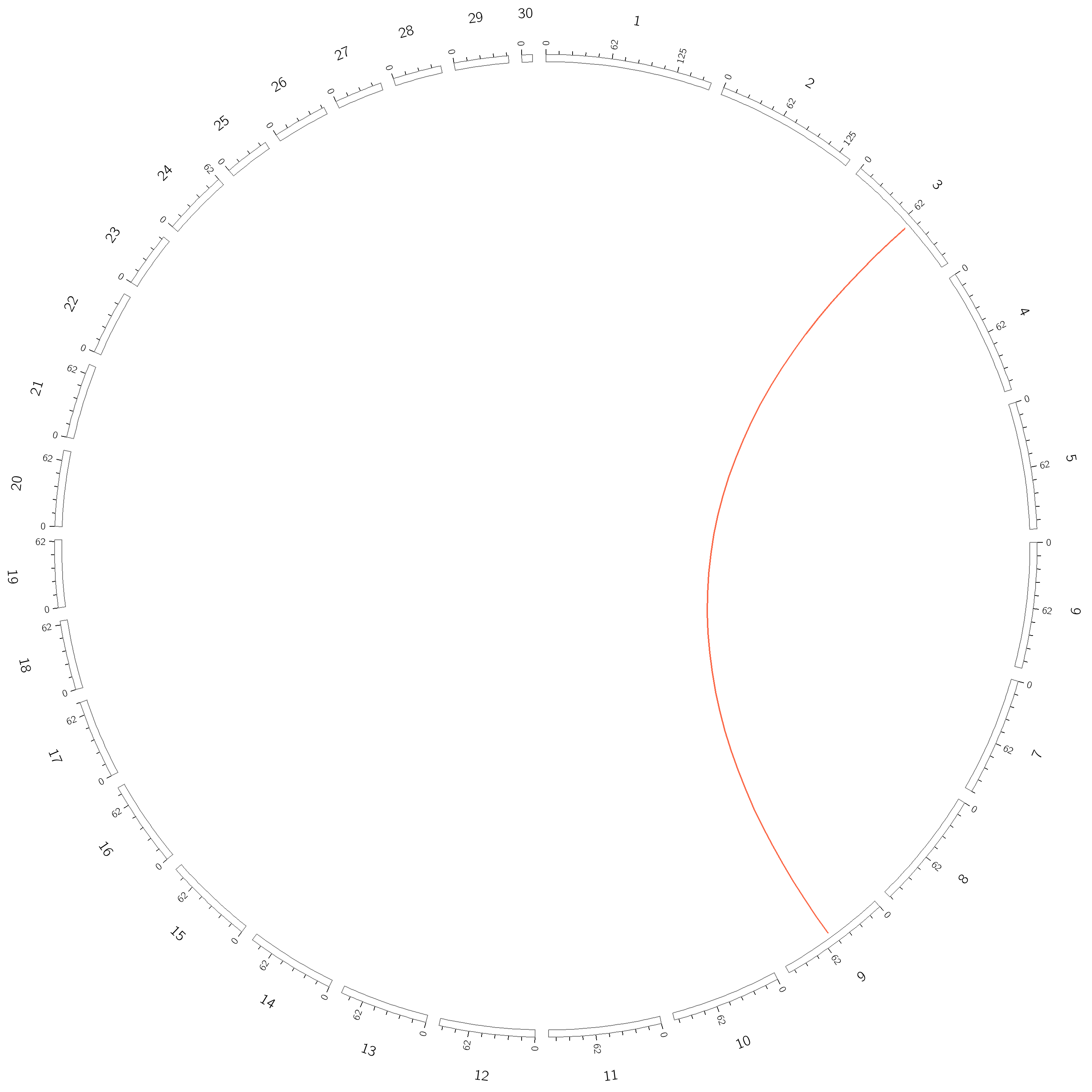

Supplement: Additional file 1: — Supplemental Data (TAGFAinteractions.xlsx, PLFAinteractions.xlsx, and CarcassInteractions.xlsx) and Figures (Circos Plots). (ZIP 22719 kb) [file 12864_2016_3235_MOESM1_ESM.zip › TAG22.png]

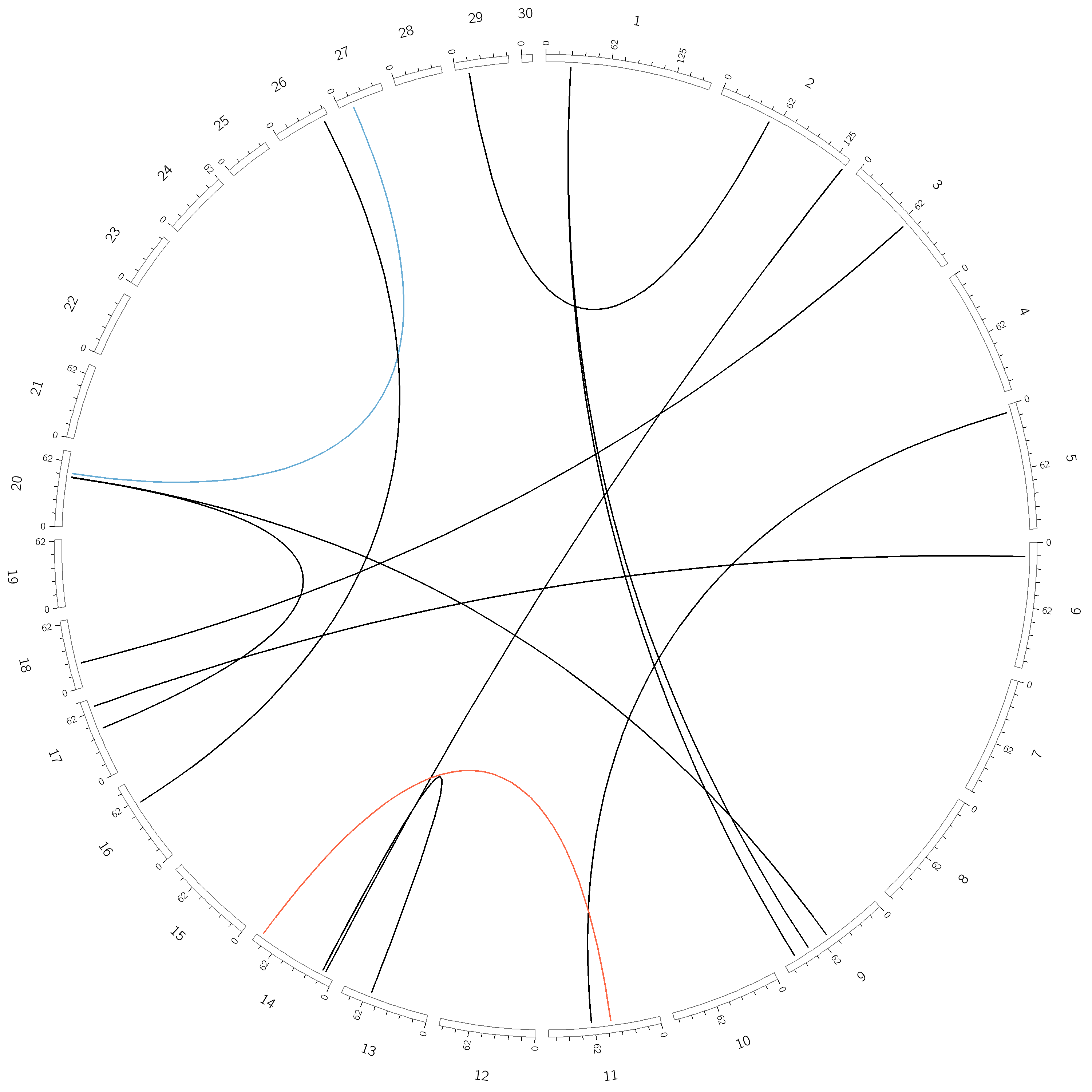

Supplement: Additional file 1: — Supplemental Data (TAGFAinteractions.xlsx, PLFAinteractions.xlsx, and CarcassInteractions.xlsx) and Figures (Circos Plots). (ZIP 22719 kb) [file 12864_2016_3235_MOESM1_ESM.zip › TAGCLAC12.png]

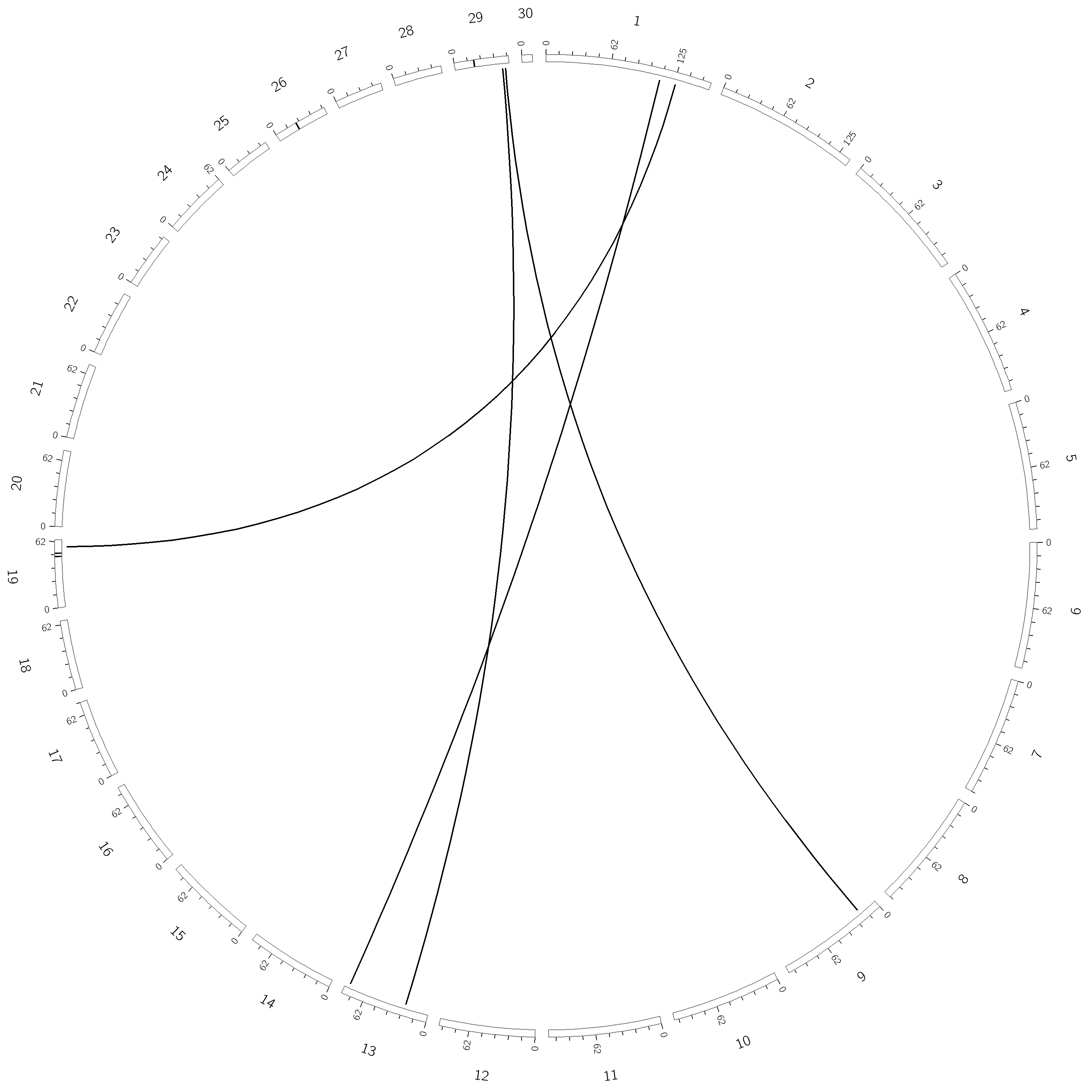

Supplement: Additional file 1: — Supplemental Data (TAGFAinteractions.xlsx, PLFAinteractions.xlsx, and CarcassInteractions.xlsx) and Figures (Circos Plots). (ZIP 22719 kb) [file 12864_2016_3235_MOESM1_ESM.zip › TAGIA.png]

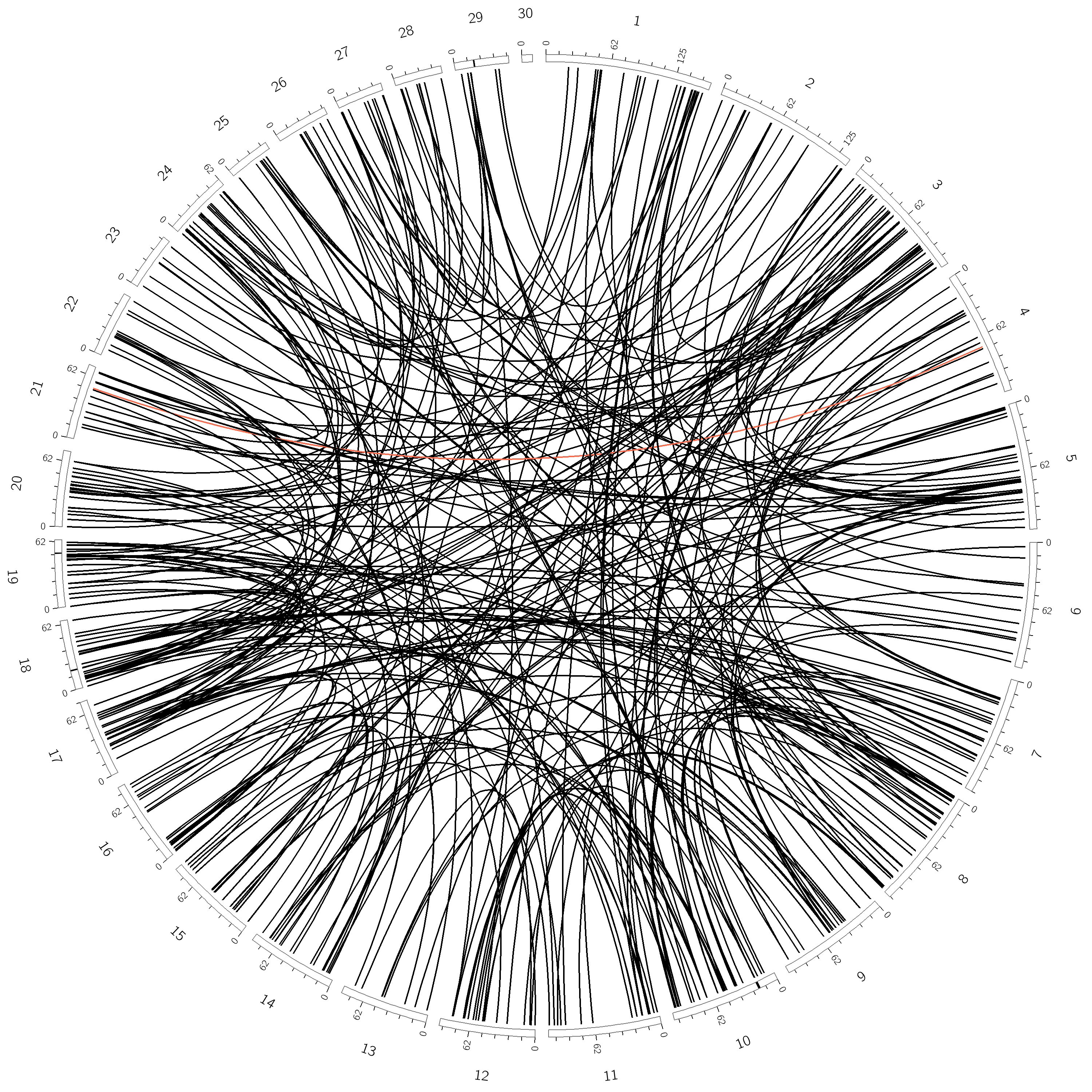

Supplement: Additional file 1: — Supplemental Data (TAGFAinteractions.xlsx, PLFAinteractions.xlsx, and CarcassInteractions.xlsx) and Figures (Circos Plots). (ZIP 22719 kb) [file 12864_2016_3235_MOESM1_ESM.zip › TAGLCFA.png]

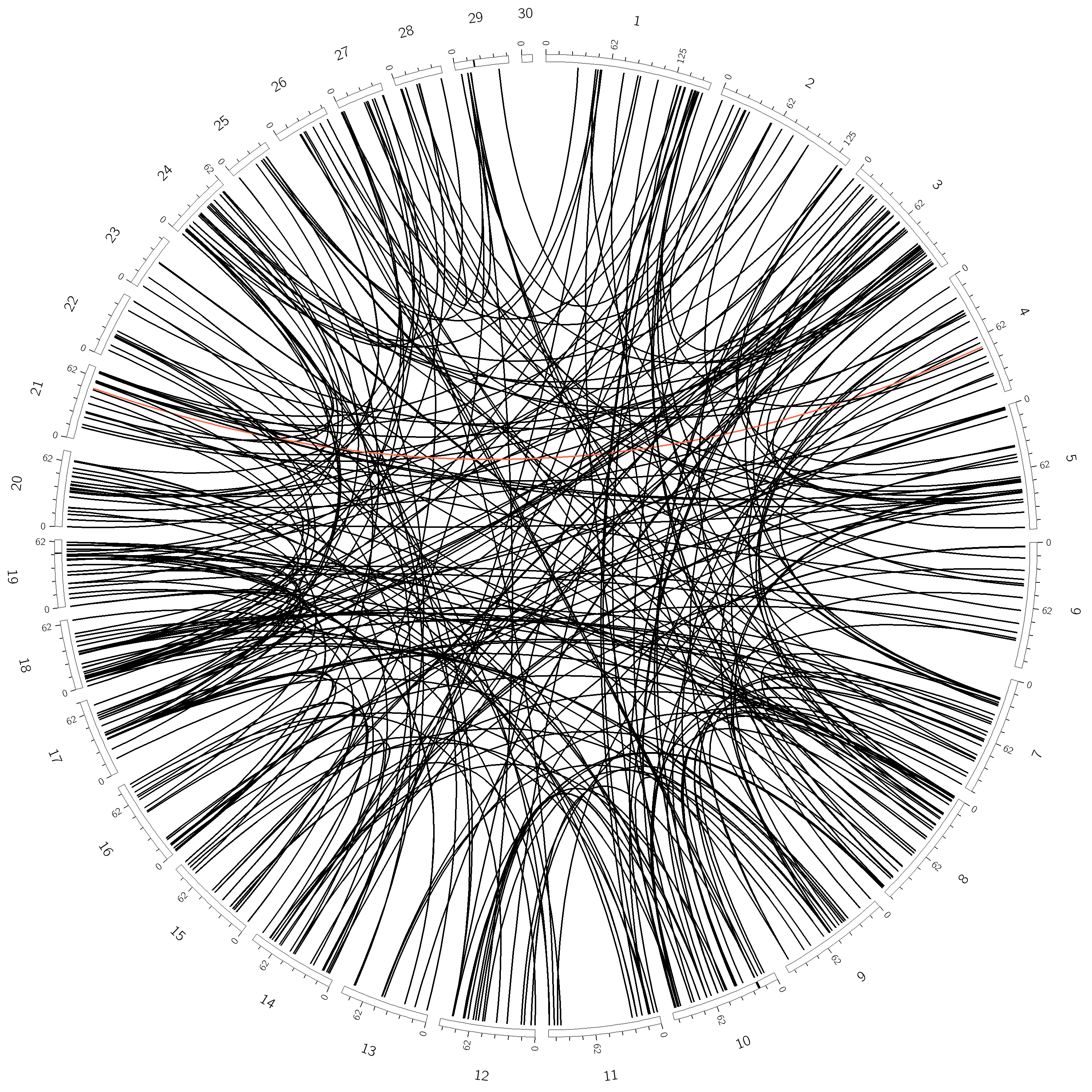

Supplement: Additional file 1: — Supplemental Data (TAGFAinteractions.xlsx, PLFAinteractions.xlsx, and CarcassInteractions.xlsx) and Figures (Circos Plots). (ZIP 22719 kb) [file 12864_2016_3235_MOESM1_ESM.zip › TAGMCFA.png]

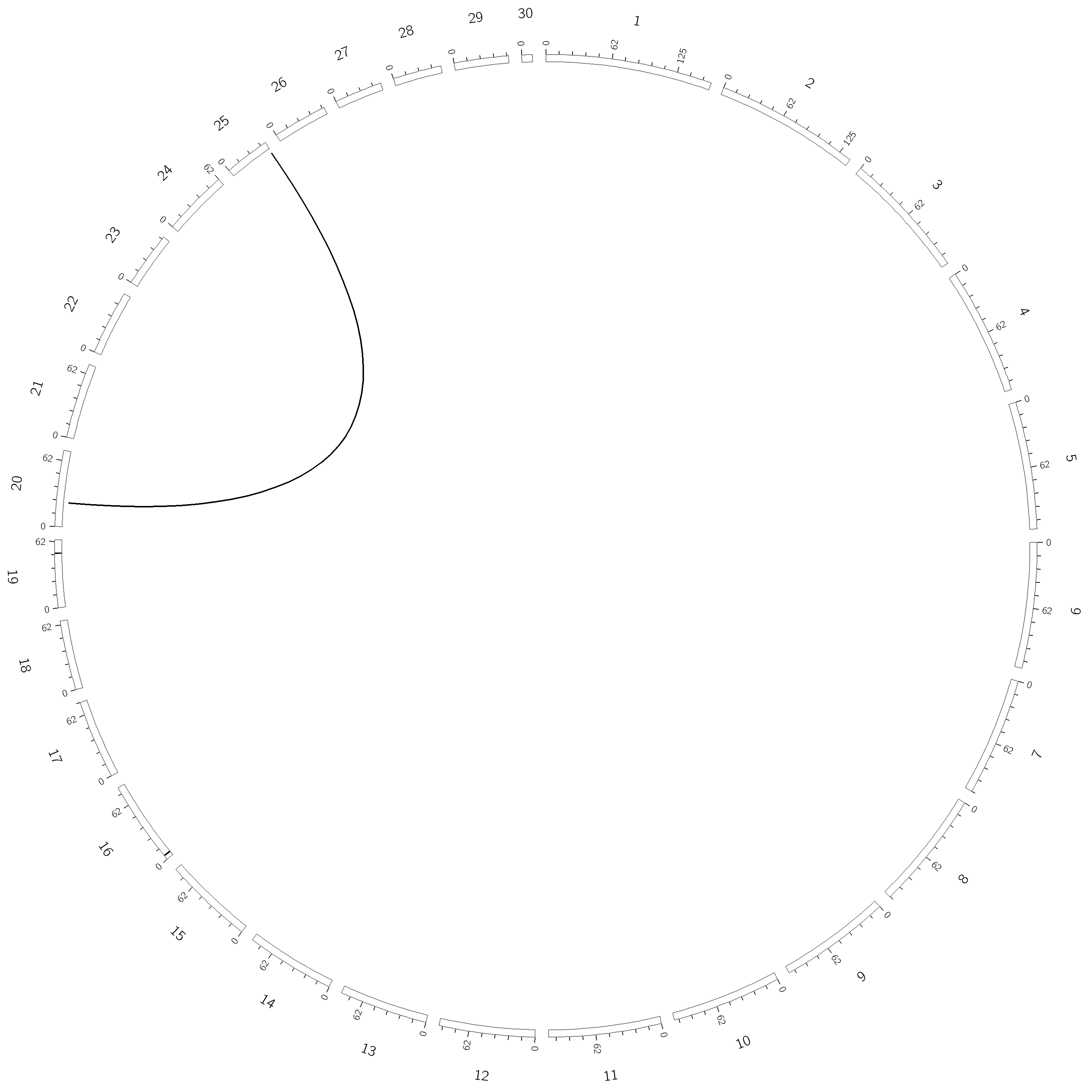

Supplement: Additional file 1: — Supplemental Data (TAGFAinteractions.xlsx, PLFAinteractions.xlsx, and CarcassInteractions.xlsx) and Figures (Circos Plots). (ZIP 22719 kb) [file 12864_2016_3235_MOESM1_ESM.zip › TAGMUFA.png]

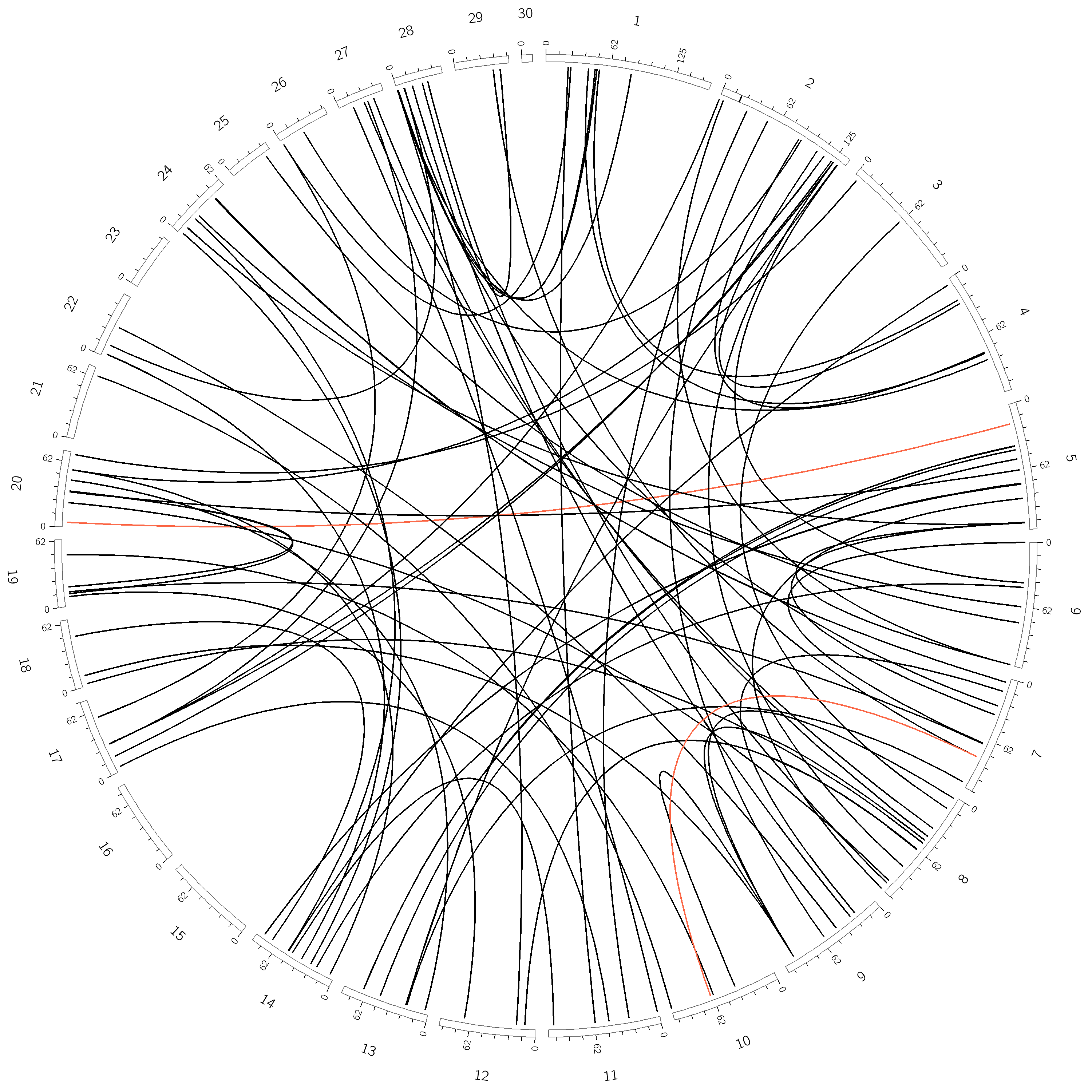

Supplement: Additional file 1: — Supplemental Data (TAGFAinteractions.xlsx, PLFAinteractions.xlsx, and CarcassInteractions.xlsx) and Figures (Circos Plots). (ZIP 22719 kb) [file 12864_2016_3235_MOESM1_ESM.zip › TAGn6.png]

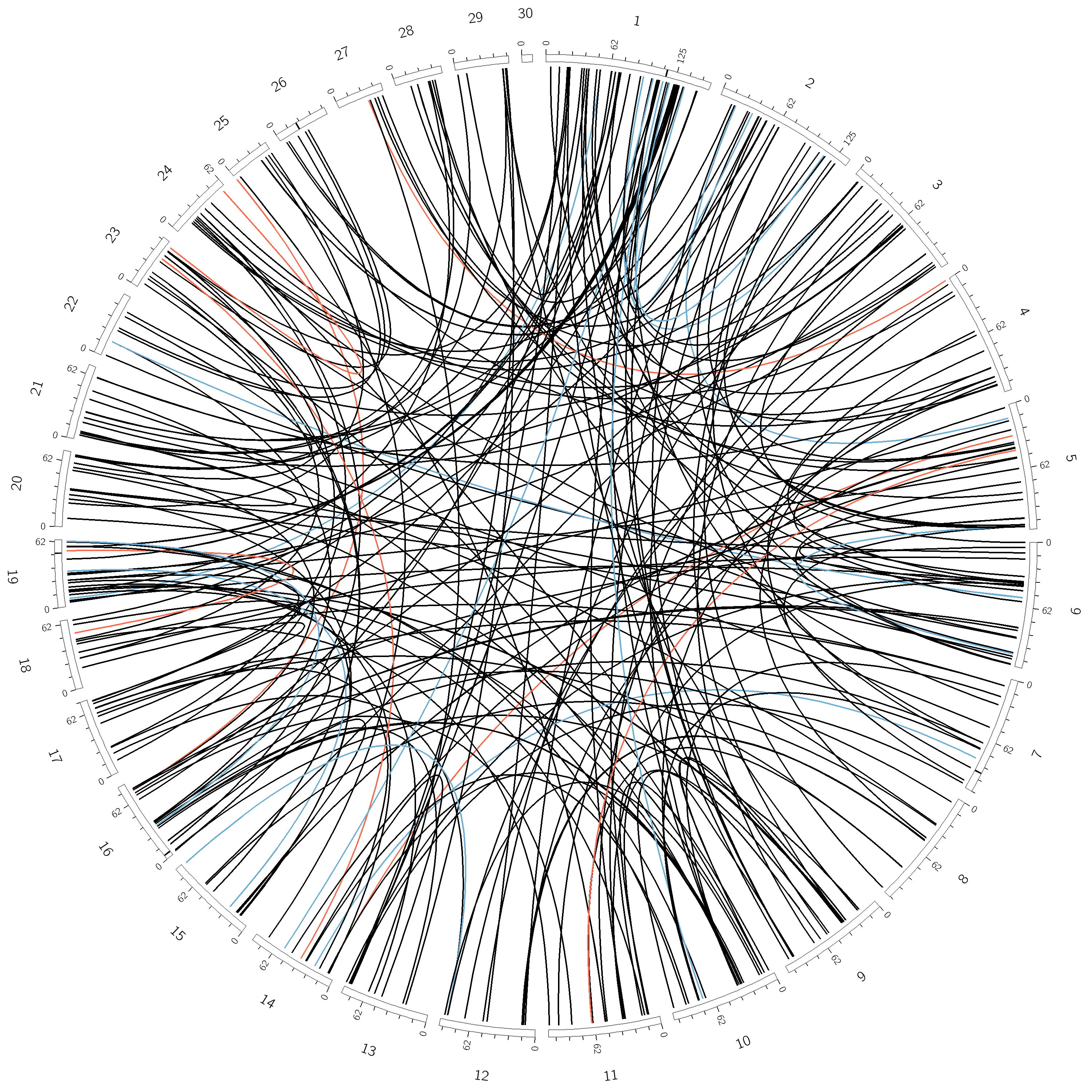

Supplement: Additional file 1: — Supplemental Data (TAGFAinteractions.xlsx, PLFAinteractions.xlsx, and CarcassInteractions.xlsx) and Figures (Circos Plots). (ZIP 22719 kb) [file 12864_2016_3235_MOESM1_ESM.zip › TAGSFA.png]

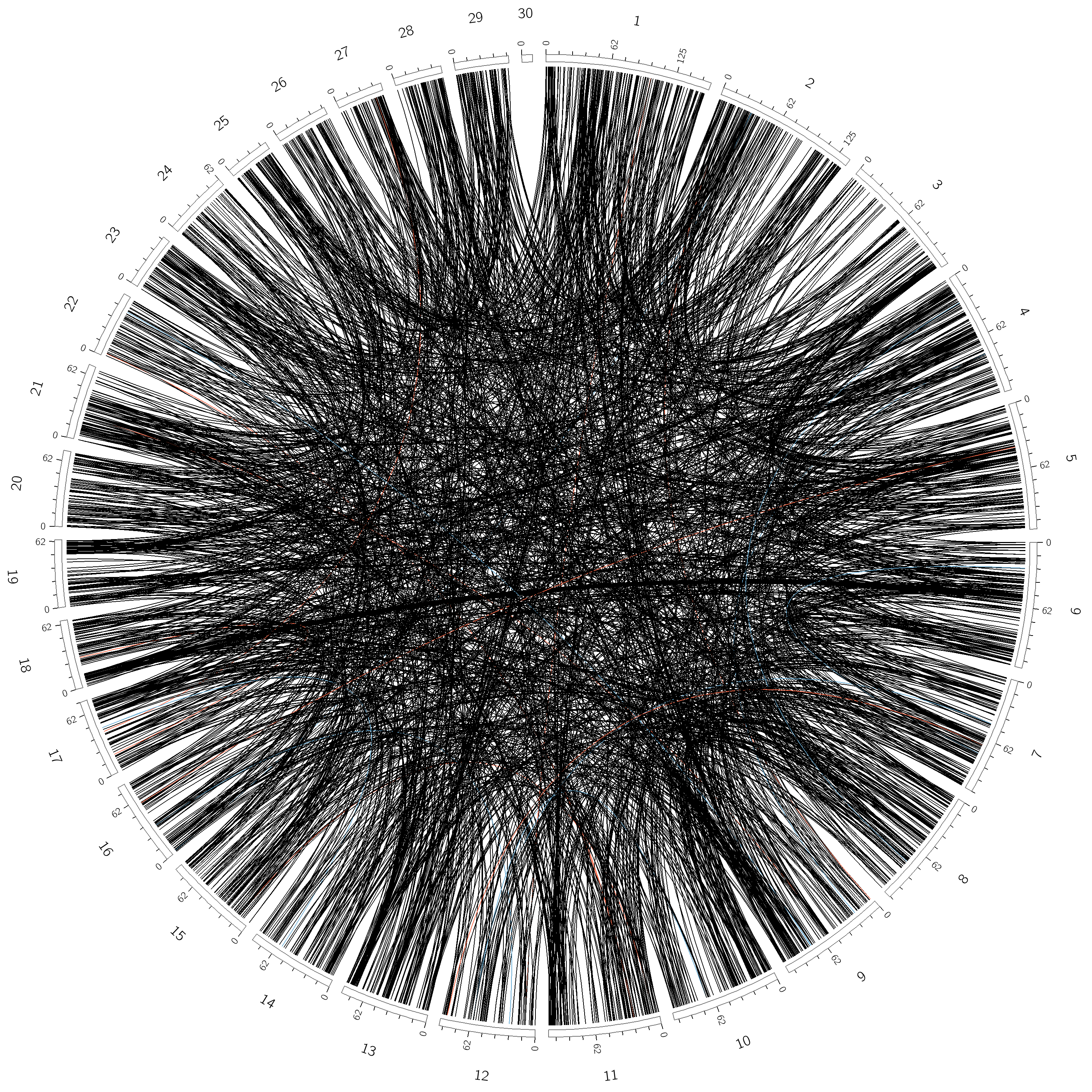

Supplement: Additional file 1: — Supplemental Data (TAGFAinteractions.xlsx, PLFAinteractions.xlsx, and CarcassInteractions.xlsx) and Figures (Circos Plots). (ZIP 22719 kb) [file 12864_2016_3235_MOESM1_ESM.zip › TAGT10T11.png]

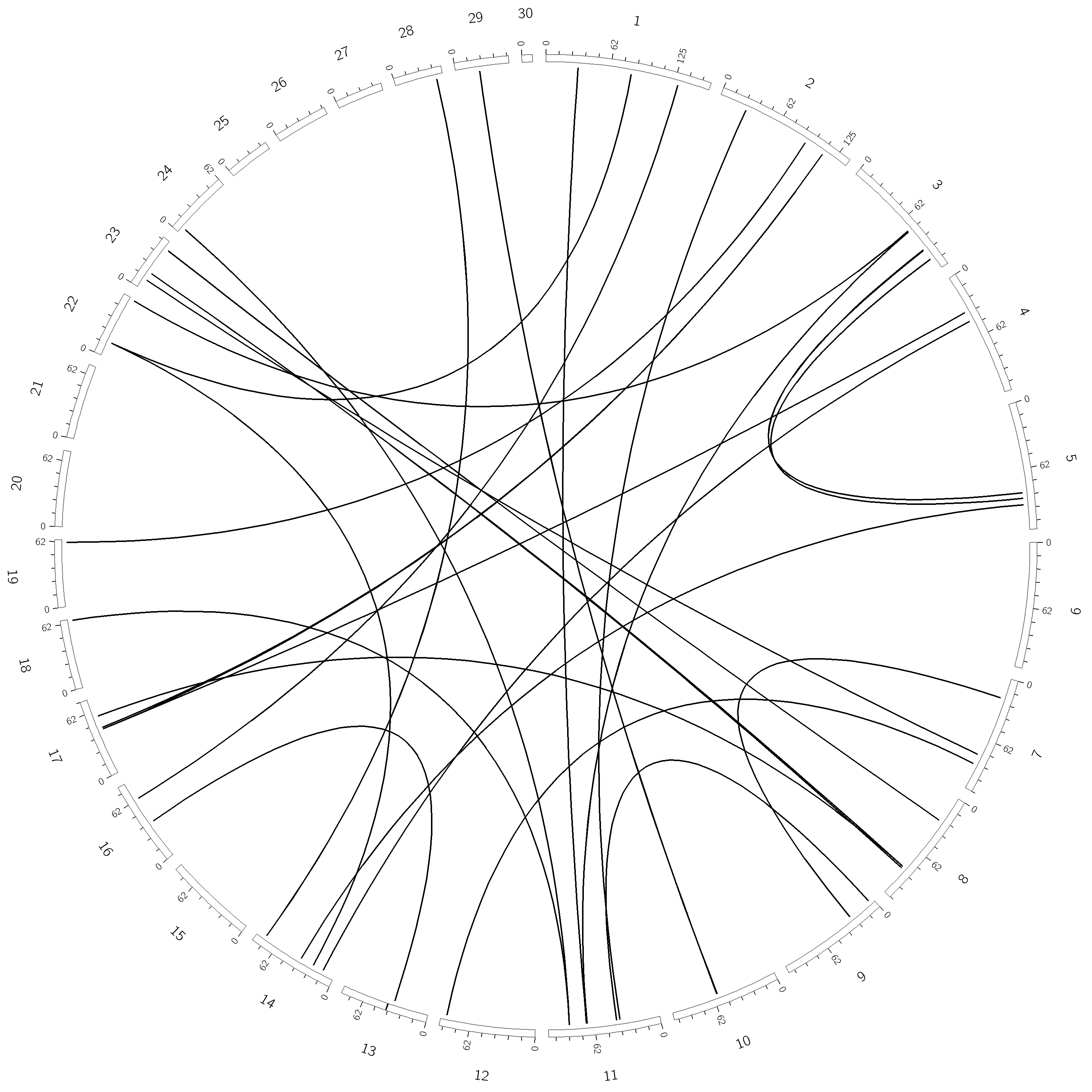

Supplement: Additional file 1: — Supplemental Data (TAGFAinteractions.xlsx, PLFAinteractions.xlsx, and CarcassInteractions.xlsx) and Figures (Circos Plots). (ZIP 22719 kb) [file 12864_2016_3235_MOESM1_ESM.zip › TAGT15.png]
